# Global atmospheric CO_2_ inverse models converging on neutral tropical land exchange, but disagreeing on fossil fuel and atmospheric growth rate

**DOI:** 10.5194/bg-16-117-2019

**Published:** 2019-01-16

**Authors:** Benjamin Gaubert, Britton B. Stephens, Sourish Basu, Frédéric Chevallier, Feng Deng, Eric A. Kort, Prabir K. Patra, Wouter Peters, Christian Rödenbeck, Tazu Saeki, David Schimel, Ingrid Van der Laan-Luijkx, Steven Wofsy, Yi Yin

**Affiliations:** 1 Atmospheric Chemistry Observations & Modeling Laboratory (ACOM), National Center for Atmospheric Research, Boulder, CO, USA; 2 Earth Observing Laboratory (EOL), National Center for Atmospheric Research, Boulder, CO, USA; 3 Earth System Research Laboratory, National Oceanic and Atmospheric Administration, Boulder, CO, USA; 4 Cooperative Institute for Research in Environmental Sciences, University of Colorado, Boulder, CO, USA; 5 Laboratoire des Sciences du Climat et de l’Environnement, Institut Pierre-Simon Laplace, CEA-CNRS-UVSQ, Gif sur Yvette, 91191 CEDEX, France; 6 Department of Physics, University of Toronto, Toronto, Canada; 7 Climate and Space Sciences and Engineering, University of Michigan, Ann Arbor, MI, USA; 8 RGGC/IACE/ACMPT, Japan Agency for Marine-Earth Science and Technology (JAMSTEC), Yokohama 236 0001, Japan; 9 Meteorology and Air Quality, Wageningen University, Wageningen, the Netherlands; 10 Max Planck Institute for Biogeochemistry, 07745 Jena, Germany; 11 Center for Global Environmental Research, National Institute for Environmental Studies, Tsukuba, Japan; 12 Jet Propulsion Laboratory, California Institute of Technology, Pasadena, CA, USA; 13 School of Engineering and Applied Science and Department of Earth and Planetary Sciences, Harvard University, Cambridge, MA, USA; 14 Division of Geological and Planetary Sciences, California Institute of Technology, Pasadena, CA, USA

## Abstract

We have compared a suite of recent global CO_2_ atmospheric inversion results to independent airborne observations and to each other, to assess their dependence on differences in northern extratropical (NET) vertical transport and to identify some of the drivers of model spread. We evaluate posterior CO_2_ concentration profiles against observations from the High-Performance Instrumented Airborne Platform for Environmental Research (HIAPER) Pole-to-Pole Observations (HIPPO) aircraft campaigns over the mid-Pacific in 2009–2011. Although the models differ in inverse approaches, assimilated observations, prior fluxes, and transport models, their broad latitudinal separation of land fluxes has converged significantly since the Atmospheric Carbon Cycle Inversion Intercomparison (TransCom 3) and the REgional Carbon Cycle Assessment and Processes (RECCAP) projects, with model spread reduced by 80% since TransCom 3 and 70% since RECCAP. Most modeled CO_2_ fields agree reasonably well with the HIPPO observations, specifically for the annual mean vertical gradients in the Northern Hemisphere. Northern Hemisphere vertical mixing no longer appears to be a dominant driver of northern versus tropical (T) annual flux differences. Our newer suite of models still gives northern extratropical land uptake that is modest relative to previous estimates (Gurney et al., 2002; Peylin et al., 2013) and near-neutral tropical land uptake for 2009–2011. Given estimates of emissions from deforestation, this implies a continued uptake in intact tropical forests that is strong relative to historical estimates (Gurney et al., 2002; Peylin et al., 2013). The results from these models for other time periods (2004–2014, 2001–2004, 1992–1996) and reevaluation of the TransCom 3 Level 2 and RECCAP results confirm that tropical land carbon fluxes including deforestation have been near neutral for several decades. However, models still have large disagreements on ocean–land partitioning. The fossil fuel (FF) and the atmospheric growth rate terms have been thought to be the best-known terms in the global carbon budget, but we show that they currently limit our ability to assess regional-scale terrestrial fluxes and ocean–land partitioning from the model ensemble.

## 1 Introduction

Current appraisals of the global atmospheric carbon budget are informed by surface fluxes computed by inverse transport models (e.g., Newsam and Enting, 1988; Tans et al., 1990; Rayner et al., 1999; Gurney et al., 2002, 2003, 2004; Peylin et al., 2013). Net carbon flux to the atmosphere is derived from temporal and spatial CO_2_ gradients given by atmospheric observations and prior estimates of component fluxes and their uncertainties. This assessment of atmospheric sources and sinks relies on (1) atmospheric tracer transport models that link fluxes to atmospheric CO_2_ fields, (2) prior emissions and sinks (e.g., from process model flux estimates), (3) the spatial and temporal representativeness and coverage of the observational network, and (4) error statistics associated with each information piece. Since the problem is underdetermined, it is essential to quantify the uncertainty and biases of posterior fluxes and CO_2_ concentrations with independent observations and cross-model comparisons. The most prominent community-wide inverse result intercomparison that included comparisons of posterior concentrations to independent observations was the TransCom 3 study (Gurney et al., 2002, 2004), which studied fluxes for the 1992–1996 period. This comparison could focus on the impact of transport model differences by optimizing the fluxes using a common method over the same regions (11 land and 11 ocean). One particular feature of the seasonally resolved (Level 2) TransCom 3 inversions (hereafter denoted as T3L2) was the direct dependence of flux estimates on vertical gradients of CO_2_ (Stephens et al., 2007), leading to a different partitioning between northern extratropical (NET) versus tropical (T) land sinks. A more recent community-wide CO_2_ inverse model intercomparison was carried out as part of the REgional Carbon Cycle Assessment and Processes project (RECCAP, http://www.globalcarbonproject.org/reccap/ last access: 7 January 2019; Canadell et al., 2011). The atmospheric inversion component of RECCAP was a comprehensive intercomparison that analyzed long-term mean, long-term trend, interannual variations, and mean seasonal variations of CO_2_ fluxes using common post-processing (Peylin et al., 2013). In RECCAP, the 11 inverse models used different inversion techniques, atmospheric models, and observational datasets. When the fluxes were analyzed for the years 2001 to 2004, Peylin et al. (2013) found an overall improved consistency between inversions on a large scale and over specific regions compared to T3L2 when the network of atmospheric sites was less dense. RECCAP inversions showed a general agreement on the total natural land carbon flux long-term mean and its interannual variability over 1991–2010. The total ocean plus land sink estimates were more robust over the NET than for the tropics and in the southern extratropics (SET). The remaining spread led to a disagreement on the NET–T–SET land partitioning, with some models simulating a stronger tropical source compensated for by larger NET and SET sinks. Peylin et al. (2013) also noted that the group of models that assimilated observations at their corresponding times rather than using monthly means had more consistent, weaker tropical sources, and weaker northern sink land fluxes.

Several additional inverse modeling intercomparison studies have more recently involved satellite, surface, and joint surface–satellite inversion (e.g., Chevallier et al., 2014; Houweling et al., 2015). In these studies, the inversion systems used space-borne retrievals of column-average dry air-mole fraction of CO_2_ (XCO_2_) from the Orbiting Carbon Observatory 2 (OCO-2) satellite since July 2014 (Eldering et al., 2017) and from the Greenhouse Gases Observing Satellite (GOSAT; Kuze et al., 2009) instrument since January 2009. Those inverse exercises, however, are still sensitive to satellite retrieval algorithms and the inversions’ prior assumptions. In particular, the results are sensitive to systematic errors from transport and satellite retrievals (Houweling et al., 2010; Chevallier, 2015).

Schimel et al. (2015) investigated the NET versus T+SET land flux partitioning as indicated by atmospheric inversions, biosphere process model simulations, and forest inventory estimates, and they estimated a large land uptake over the tropics by intact forests due to a significant CO_2_ fertilization effect. This study argued for the importance of comparing posterior CO_2_ fields to observations, which was not done in RECCAP, in order to fully understand and predict terrestrial land sinks, as well as their variation due to CO_2_ and climate feedbacks. A follow-up inversion intercomparison focused on East Asia and found that large flux adjustments were possible even though models simulated the observed gradient in vertical profiles measured by aircraft well, because the uncertainties from model transport and fossil fuel (FF) prior emissions were compensated for by the flux adjustments (Thompson et al., 2016).

The HIAPER Pole-to-Pole Observaions (HIPPO) campaign (Wofsy, 2011, https://doi.org/10.3334/CDIAC/HIPPO_010) spanned large latitudinal, vertical, and temporal coverage (2009 to 2011) and provides a useful atmospheric trace gas dataset for investigating the consistency of inverse fluxes and posterior concentration results. Graven et al. (2013) found an increase in the CO_2_ seasonal amplitude by up to 50% at mid- to high latitudes of the Northern Hemisphere and at altitudes ranging between 3 and 6 km between the HIPPO period and the 1950s. Deng et al. (2015) compared posterior CO_2_ and O_3_ fields from GEOS-Chem to the HIPPO observations to diagnose the impact of the upper troposphere and lower stratosphere (UTLS) definition on retrieved fluxes. These results indicate a significant impact of transport errors on retrieved fluxes. Frankenberg et al. (2016) evaluated the CarbonTracker CT2013B and Monitoring Atmospheric Composition and Climate (MACC) v13r1 atmospheric inverse models, as well as satellite retrievals from GOSAT, TES (Tropospheric Emission Spectrometer), and AIRS (Atmospheric Infrared Sounder) in comparison to HIPPO measurements. They found that, despite an overall agreement between inversions and HIPPO measurements, systematic model transport errors remain important.

After years of continuous model development, the goal of this study is to investigate whether global inverse models are still highly dependent on Northern Hemisphere vertical transport errors and on prior flux estimates and their uncertainties used in the inversions.

Our two main approaches to answer this question are described as follows.

- First, we compare modeled CO_2_ after flux optimization to independent aircraft in situ CO_2_ observations from the HIPPO campaign (2009–2011).
- Second, we compare the observationally constrained fluxes across models and to budget estimates provided by the Global Carbon budget 2016 (hereafter denoted GCP2016; Le Quéré et al., 2016), both for latitudinal bands and on a global scale.

Measurements and inversion systems are described in Sect. 2. In Sect. 3.1, we present the results of the comparison of modeled posterior CO_2_ vertical gradients with HIPPO measurements. In Sect. 3.2, we analyze the differences in the meridional distribution of land sinks and global carbon estimates for the years 2009 to 2011 from inverse modeling of atmospheric in situ observations together and with GCP2016. In Sect. 3.3, we compare the inverse model and GCP2016 estimates at the global scale, including prescribed fossil and retrieved atmospheric growth rate terms. Conclusions and a summary of the findings are given in Sect. 4.

## 2 Methods

### 2.1 Participating models

The list of participating inverse models is shown in Table 1 and more details are available in the Supplement. These include 10 different inverse modeling systems or system variants. The inversion systems differ in many aspects such as transport models, wind fields, analysis procedures, and subset of assimilated observations. The atmospheric chemistry-transport model (ACTM) system performed two inversions with different prescribed fossil fuel emissions (Saeki and Patra, 2017), one based on totals from the Carbon Dioxide Information Analysis Center (CDIAC; Boden et al., 2016) and another based on the International Energy Agency (IEA/OECD, 2016), which allows us to assess sensitivity to the FF prior only. This is also the case for the two CarbonTracker Europe versions, CTE2016-FT (Fast Track) and CTE2017-FT, where only the subset of observations and the FF prior are different (van der Laan-Luijkx et al., 2017).

It is worth noting that some inverse models are constructed in a similar framework. Some share the same transport model, such as TM5 that is used in four inversions, and some use the same meteorological fields. Five inverse systems nudge their forecast field to the ERA-Interim reanalysis (Dee et al., 2011). The two longest flux estimates, from CAMS (v16r1) and Jena (s85_v4.1), are used to reproduce the comparison with observations as in Stephens et al. (2007) over the T3L2 period (1992 to 1996). The Jena s85_v4.1 and s04_v4.1 inversions differ in their calculation periods and station sets used: Jena s85_v4.1 starts in 1985 using only 23 stations that cover this entire period, while s04_v4.1 uses many more sites (59) and starts in 2004. This also allows us to separate the impact of the number of sites assimilated over the most recent period.

### 2.2 The Global Carbon Budget 2016

The Global Carbon Project (GCP) gathers observational and model-based flux estimates from multiple organizations and research groups around the world to yearly report a global budget of atmospheric CO_2_ (Le Quéré et al., 2016). GCP2016 is the most recent version with flux estimates forced to balance globally. The most recent version (GCP2017; Le Quéré et al., 2018) separated an explicit unknown ocean or land flux term, which prevents simple comparisons of the type presented here. Specifically, the land–ocean partitioning in GCP2016 is based on multiple observational constraints on the ocean flux for the 1990s, extrapolated forward with a suite of seven global ocean models. As pointed out in Le Quéré et al. (2018), there are considerable uncertainties in this extrapolation, with the estimated ocean–land partitioning for later decades dependent on the models. The GCP2016 atmospheric growth rate is derived from atmospheric CO_2_ measurements at marine boundary layer (MBL) sites made by the US National Oceanic and Atmospheric Administration (NOAA) Earth System Research Laboratory (ESRL; Masarie and Tans, 1995; Dlugokencky and Tans, 2018). CO_2_ emissions from land-use change (ELUC) are the net sum of all anthropogenic activities: deforestation, afforestation, logging, and shifting cultivation. Total emissions are estimated, following the bookkeeping method (Houghton, 2003; Houghton et al., 2012), with complementary interannual variability calculated from satellite data when available (van der Werf et al., 2010; Giglio et al., 2013). The average ELUC for the year 2009 to 2011 included here is estimated to be 0.85 PgC yr^−1^ with an uncertainty of 0.5PgC yr^−1^. These emissions are added to the GCP2016 land sink for comparison to atmospheric inversion estimates.

Finally, the land sink is estimated in GCP2016 as a residual from all other components of the carbon budget. The GCP2016 method treats the riverine flux of carbon from land to ocean to atmosphere as separate components of the total air–land and air–sea fluxes and subtracts an estimate of this flux (0.45 PgC yr^−1^; Jacobson et al., 2007) from the pCO2-based sea-to-air flux estimates to match estimates of the anthropogenic ocean sink alone. Because the land sink is a residual, this increase in the magnitude of the ocean sink results in a corresponding reduction by 0.45 PgC yr^−1^ in the magnitude of the land sink in GCP2016. To compare to atmospheric inverse flux estimates, which represent the total air–sea and air–land fluxes, we have adjusted the GCP2016 ocean and land flux estimates by this same 0.45 PgC yr^−1^, decreasing the ocean sink and increasing the land sink.

Note that we do not show GCP2016 estimates here as a truth metric against which to evaluate the models, but rather as one estimate of an internally consistent global budget that provides a useful reference for exploring axes of variability in our models and comparing to other community estimates.

### 2.3 HIPPO observations and fitting procedures

**Figure 1.**
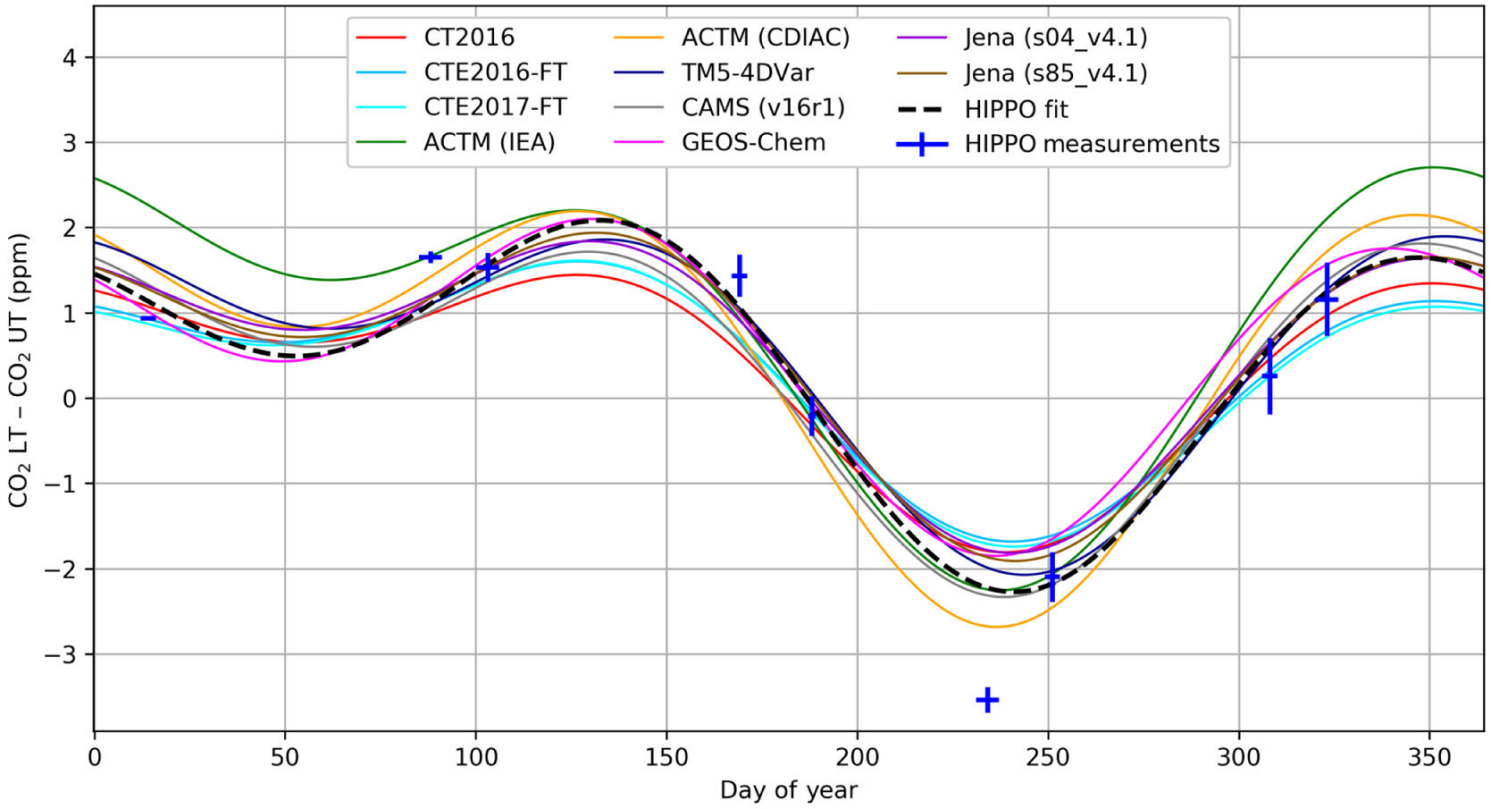
Reconstructed annual cycle in northern extratropical vertical CO_2_ gradients, obtained from fits using two harmonics of the HIPPO data and correspondingly sampled model outputs, averaged over 20 to 90° N (1000 to 800 hPa minus 800 to 400 hPa). The CO_2_ average curtain observations for each of nine atmospheric transects have been added on the graph to illustrate the data uncertainties and temporal coverage, the *y*-axis error bar is derived from the range of disagreement among the three in situ instruments on board (QCLS, OMS, and AO2; see Supplement), and the line average is derived from the CO2.X merged dataset. The horizontal whiskers represent the time span of the flights contributing to each average. The observed line shown here is not a direct fit to the observation points, but rather comes from an average of fits to individual 100 hPa by 5° latitude bins as described in the text.

The HIPPO project (Wofsy, 2011) used the NSF/NCAR Gulfstream V aircraft (GV) to conduct 5-month-long campaigns in different seasons over 3 years (2009–2011; see Supplement) that consisted of vertical profiling along North–South-Pacific transects between 87° N and 67° S. The five campaigns included nine transects of the NET Pacific. We exclude observations over North America conducted between Colorado and Alaska (Fig. S1 in the Supplement). HIPPO flew three different in situ CO_2_ instruments and two whole air samplers with laboratory CO_2_ measurements. We use the recommended CO2.X variable which comes primarily from the Harvard quantum cascade laser spectrometer (QCLS), gap filled during calibration sequences, and compare to the other systems to constrain potential systematic biases (see Supplement). We calculate the NET vertical gradient as the difference between the average from 1000 to 800 hPa for the lower troposphere (LT) and the average from 800 to 400 hPa for the upper troposphere (UT), spanning the latitude range from 20 to 90° N. To do this, we first detrend the observations and model sampled along the flight-track output by subtracting a deseasonalized and smoothed long-term trend record from the fit of the Mauna Loa Observatory in situ measurement time series to provide a common reference for both observations and models, and we bin the observations by 100 hPa in pressure and 5° in latitude bins. We then fit each bin with a curve using two harmonics and constant offset, and we average the resulting fits across boxes and pressure levels, with latitude weighting (see Supplement). Figure 1 shows the resulting daily fit of the annual cycle for the HIPPO observations and model simulations of the NET vertical gradient. Qualitatively, it shows that most models reproduce the CO_2_ cycle well, with positive gradients in winter over a broad peak and negative gradients in summer over a narrower trough. The three CarbonTracker inversions (CT2016, CTE2016-FT, and CTE2017-FT) have somewhat lower seasonal gradient amplitude, while the two ACTM inversions (ACTM-IEA and ACTM-CDIAC) show larger amplitude. More quantitative details are given in Sect. 3.1. To illustrate the temporal coverage of the observations, we plot the measurements of the nine HIPPO transects in Fig. 1 as simple differences of the latitude-weighted average concentrations within the LT and UT boxes for each transect, while an example of a fit to an individual bin is shown in Fig. S1.

The QCLS instrument has a 1*σ* precision of 20 ppb (Santoni et al., 2014), and for all five CO_2_ systems on the GV the instrumental precision is negligible for the large-scale average metrics we present here. More relevant sources of uncertainty are associated with the potential for altitude-dependent biases that might result from inlet or cabin-pressure effects, as well as misrepresentation of synoptic transport in the models. We estimate (i) uncertainty in the annual-mean NET vertical gradient metric by comparison of the five independent instruments and whole air samplers to be ± 0.15 ppm (see Supplement) and (ii) uncertainty on the individual HIPPO transect values to range from 0.02 to 0.48 ppm as shown by the vertical bars in Fig. 1. These values are derived from the maximum absolute differences between the sensors, which we conservatively treat as best-guess 1*σ* uncertainty estimates. These uncertainty estimates correspond to the vertical gradient as observed by the HIPPO flight tracks and calculated with the fitting procedure used here. Because we use model output along the flight tracks and treat model output and observations identically in our calculations, we do not include an estimate of potential spatial sampling bias, but we do use model output to assess the spatial representativeness of our calculated metrics with respect to full 150° W transect and full zonal means in Sect. 4 of the Supplement (Figs. S5, S6). Also, because the models are driven by reanalysis winds, they should capture the position of synoptic systems and associated transport. However, the wind fields and model transport may be biased, which could result in different vertical gradients for reasons unrelated to the fluxes of interest. We have estimated synoptic variability in the vertical gradient metric and find a worst-case potential model synoptic sampling bias of ±0.06 ppm for the annual mean, ±0.14 ppm for JFM, and ±0.15 ppm for JAS (1*σ*; see Supplement).

## 3 Results

### 3.1 Fluxes and posterior CO_2_ comparisons with HIPPO

Each individual inversion system adjusts fluxes to fit the concentration fields with its given transport scheme and a priori source and sink information. Biases can appear in the retrieved posterior CO_2_ resulting from errors in the estimated fluxes or from specific biases in transport to the location of the independent data (here in particular vertical transport to the upper atmosphere). We first evaluate if the spread of retrieved land fluxes over different zonal bands is correlated with NET vertical CO_2_ gradients and if the modeled gradients match observations, as was previously done for the T3L2 models by Stephens et al. (2007).

**Figure 2.**
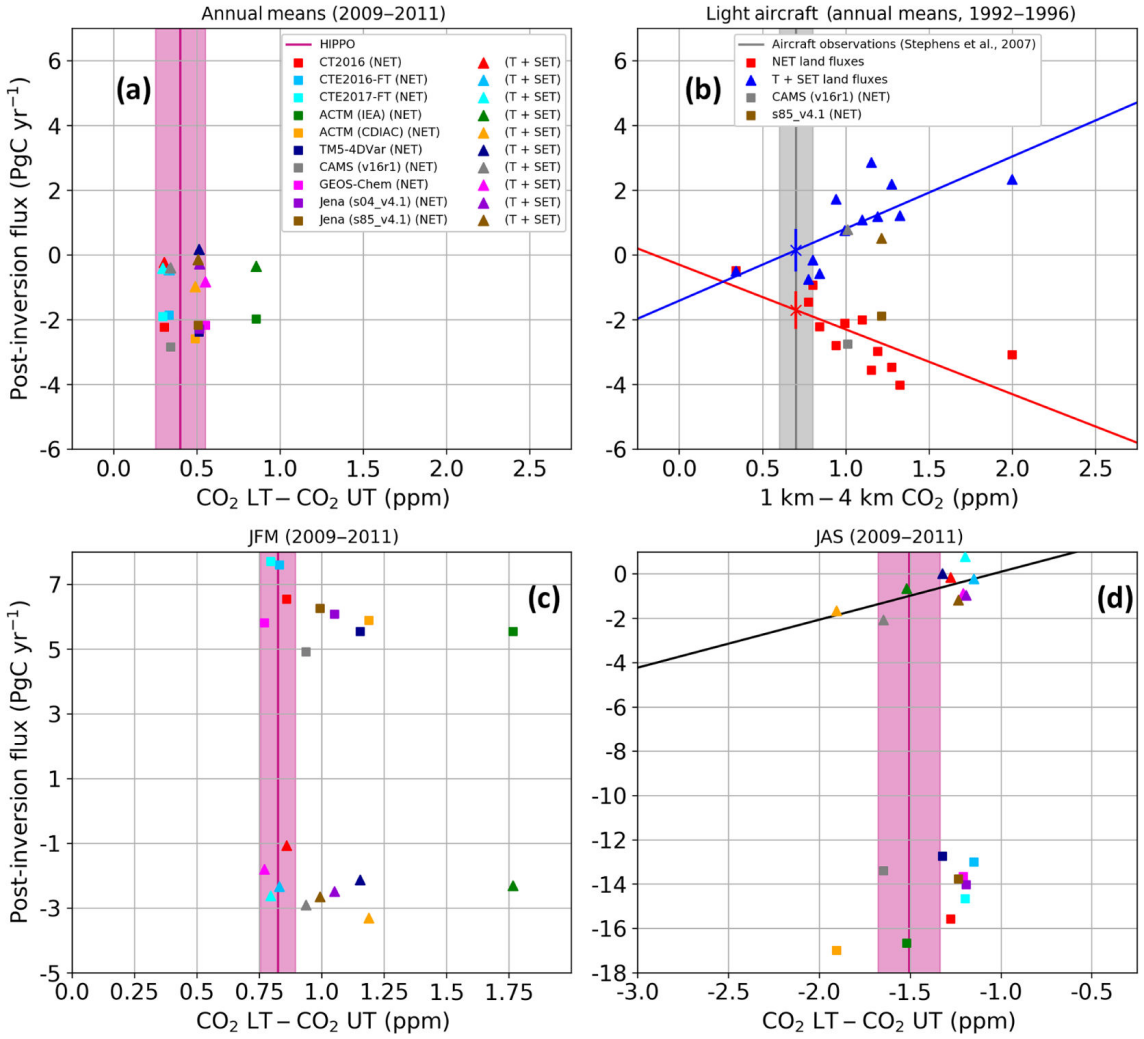
Retrieved fluxes versus NET vertical gradients. **(a)** Annual mean NET land and T+SET land fluxes versus posterior NET vertical gradients (lower minus upper troposphere) from model output along HIPPO flight tracks and HIPPO observations (pink line) for the period 2009–2011. The shaded area represents an estimate of measurement uncertainty of ±0.15 ppm for the annual mean, as estimated in the Sect. S2 in the Supplement. Inverse model posterior concentration gradients and fluxes are shown as points (squares represent NET; triangles represent T+SET). The vertical axis represents the integrated annual mean land fluxes (PgC yr^−1^). **(b)** Same as **(a)** but for 1992–1996 and showing TransCom 3 Level 2 models and our two current models that span this time period, showing dependence of posterior fluxes on transport and a large range of transport biases. Annual mean NET (red squares) and T+SET (blue triangles) land carbon fluxes for 1992–1996 estimated by the 12 T3L2 models plotted as a function of the models’ post-inversion predicted mean vertical CO_2_ gradients at 10 light-aircraft profiling sites (adapted from Stephens et al., 2007) with fluxes partitioned by TransCom region. The Jena (s85_v4.1) and the CAMS (v16r1) simulations have also been sampled at the same light-aircraft locations but their fluxes are partitioned at 20° N and 20° S. The crosses show our new best estimate of the fluxes estimated by the regression of all T3L2 models. The error bars on these points are estimated using the standard error of the regressions. **(c)** Same as panel **(a)** for January–February–March (JFM), and **(d)** same as panel **(a)** for July–August–September (JAS). For the seasonal plots, the width of the pink bar is 0.07 ppm for JFM and 0.17 for JAS. In panel **(d)**, the black line represents the regression line, shown because the relationship is statistically significant at a 95% confidence interval.

Figure 2a presents the results for the HIPPO and model vertical gradients and model fluxes, broken into NET and T+SET regions for the years 2009–2011. The mean and relative spread of 10 simulations for the posterior annual mean NET land flux is −2.24PgC yr^−1^ ±0.29PgC yr^−1^ (13 %, 1*σ*). Aside from the ACTM-IEA simulation, all models are within the uncertainty range of 0.15 ppm or 50% of the measured vertical gradient. This contrasts to the TransCom 3 Level 2 simulations which had an annual mean of −2.42PgC yr^−1^ ±1.05 (43%)PgC yr^−1^ for NET land flux and disagreed with the observed vertical gradient by ~ 0.5 ppm on average and as much as 1.3 ppm (186 %). As listed in Table 1, the inversions have significant differences in transport model, resolution, and driving meteorology and are converging despite these differences. In addition, there are no apparent relationships between vertical gradients and NET nor T+SET land fluxes. The standard deviation across 10 simulations on the difference between NET land and T+SET is 0.4 PgC yr^−1^ while it was 2.1 PgC yr^−1^ in T3L2 (Gurney et al., 2004; Gurney and Denning, 2013) and 1.28 PgC yr^−1^ in RECCAP (Peylin et al., 2013), representing a steady and dramatic convergence of model estimates over the past 15 years. We reproduce the Stephens et al. (2007) annual mean figure in Fig. 2b, with the exception of showing T+SET instead of T, to highlight those differences. It is important to note that these results correspond to a different period and different models, with a smaller network of assimilated in situ network measurements and assimilation of monthly mean rather than discrete measurements. We took advantage of the two models that span the 1992–1996 period, CAMS (v16r1) and Jena (s85_v4.1), to further investigate differences from the T3L2 period. Those two models are quite close to the 2009–2011 vertical gradient observations (Fig. 2a), but they both overestimate the 1992–1996 vertical gradients (Fig. 2b). Notably, they fall along the lines fit to the T3L2 models in Fig. 2b, which could be a coincidence, but might also suggest that despite agreeing with the other models on the latitudinal flux distribution for 2009–2011 these models overestimate tropical sources and northern sinks during 1992–1996. This would require that these models be more dependent on vertical mixing biases in the earlier period. The different number of assimilated sites is one potential factor that might explain different biases in retrieved fluxes for these two periods, but this is not seen for the comparison of the two versions of the Jena model assimilating different numbers of sites during 2009–2011. It is worth noting that reanalyses of meteorological observations have noticeably improved thanks to a better representation of unresolved processes in global models, improved data assimilation methods, and the increasing availability of satellite data, which makes the reanalyses perform better in the 2000s than for the 1990s and earlier (e.g., Gelaro et al., 2017; Bauer et al., 2015). As an example, the assimilation of new observations from the constellation of COSMIC global positioning system radio occultation (GPSRO) satellites has led to a significant improvement in meteorological analyses and forecasts (e.g., Healy, 2008).

One concern is the spatial representativeness of the HIPPO measurements which were made over the Pacific Ocean while the light-aircraft observations used by Stephens et al. (2007) were mostly measuring profiles over land. We discuss this issue in the Supplement and show that across models HIPPO vertical gradients are significantly representative of the zonal mean for the 3-year mean and every year individually (Fig. S5). Seasonally (Fig. S6), it appears that the vertical gradients are representative of the parallel 150W for winter (JFM), spring (AMJ), and fall (OND) seasons, representative of the zonal mean for winter (JFM) and fall (OND), and representative of the zonal average over land only in boreal summer (JAS). We did find a significant correlation between vertical gradients defined by the HIPPO flight tracks and land zonal means during summer (JAS), when vertical gradients are weak.

**Figure 3.**
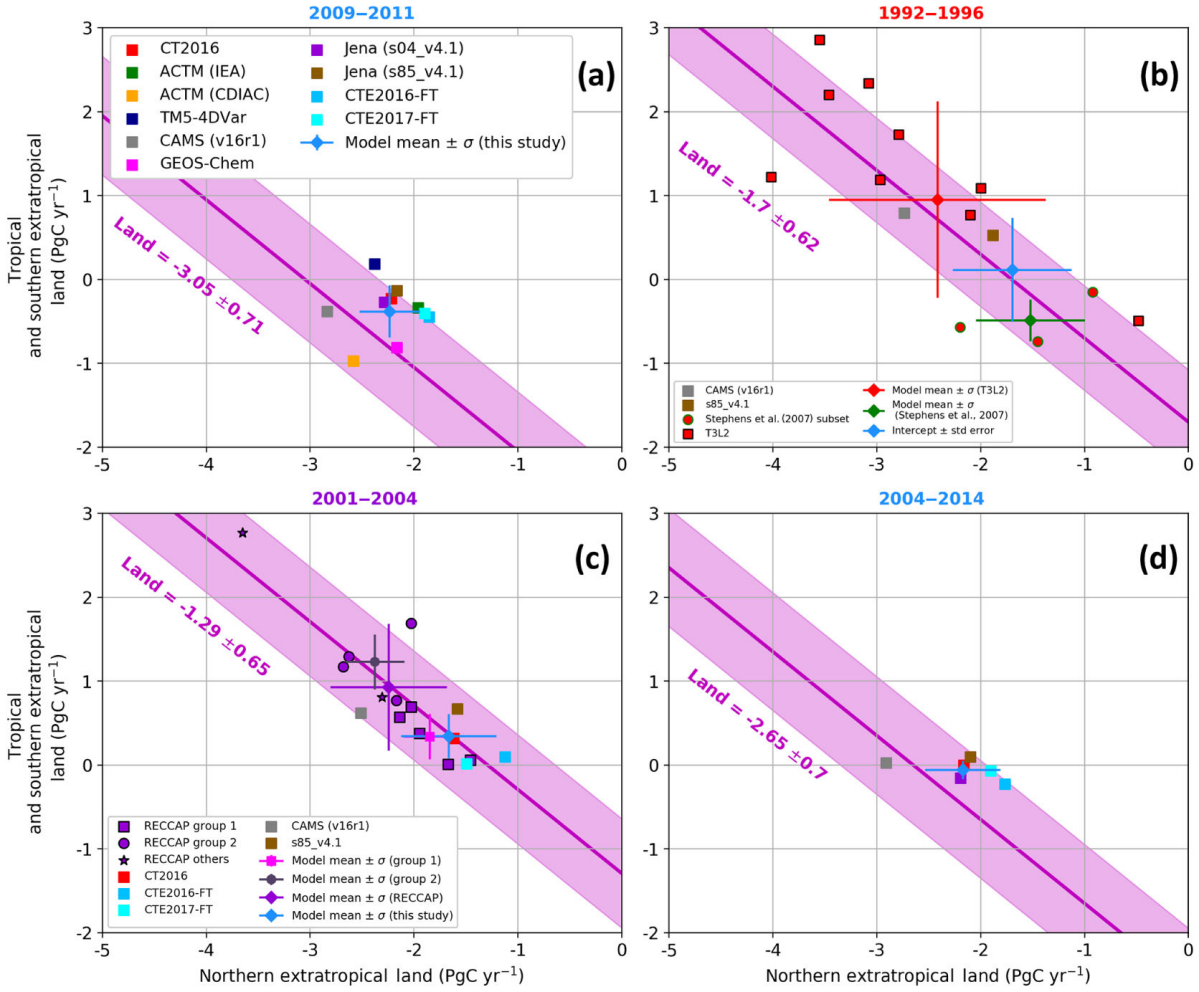
Tropical and southern extratropical (T+SET) versus northern extratropical (NET) land fluxes for the periods **(a)** 2009–2011, **(b)** 1992–1996, **(c)** 2001–2004, and **(d)**2004–2014. The new models used in this study are represented by squares and the average of the available or selected simulations is shown in blue with 1 standard deviation error bars. The pink line and shaded area represents the GCP2016 (river adjusted) estimates of the total land sink for the given period. **(a)** Results for the HIPPO period 2009–2011; **(b)** results for the T3L2 period 1992–1996. The TransCom 3 Level 2 outputs (Gurney et al., 2004) are shown in red, with the vertical gradient selected models from Stephens et al. (2007) as circles outlined in green and the rest as red squares outlined in black. The intercept of the regression line with the observed vertical gradient (Fig. 2) is used to define our best flux estimate with error bars estimated by the standard error of the linear regression. **(c)** Results for the RECCAP period 2001–2004. Also, from Peylin et al. (2013), model means and standard deviations are shown in pink for the subgroup 1 (Jena, LSCEa, MACC-II, CTE2013, CT2011_oi) and in gray for the subgroup 2 (MATCH, CCAM, TrC, NICAM). Panel **(d)** shows the results from our new set of models for the period 2004–2014.

**Figure 4.**
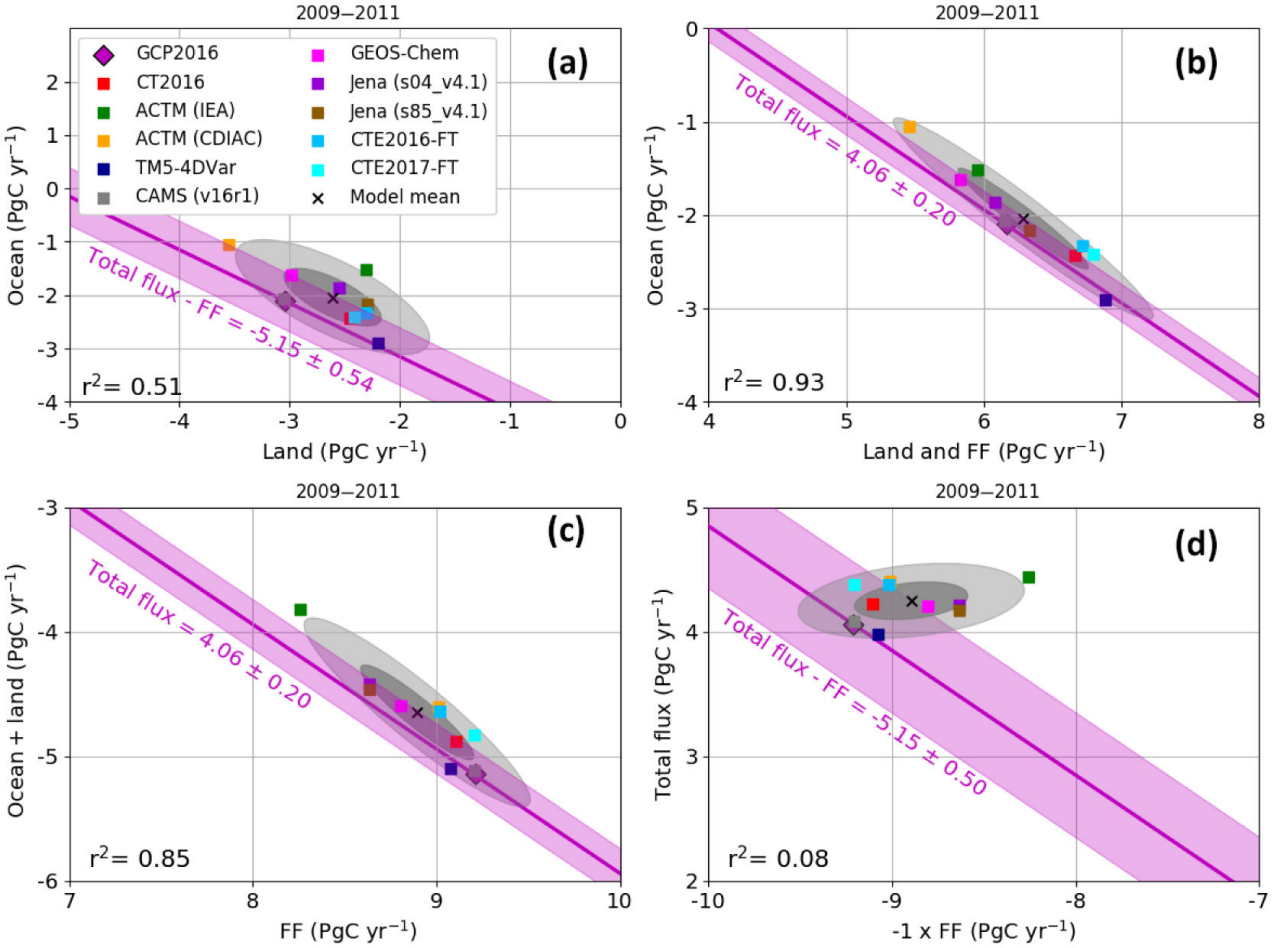
Synthesis of globally integrated fluxes for the 2009–2011 period, in PgC yr^−1^. Each inversion is represented by a square and the model mean by a × symbol. The GCP2016 estimates are a pink diamond, which is sometimes hard to see because it is superimposed in each panel by the gray CAMS point. We have adjusted the GCP2016 ocean and land flux estimates by the riverine flux of carbon from land to ocean to atmosphere (0.45 PgC yr^−1^; Jacobson et al., 2007; Le Quéré et al., 2018), decreasing the ocean sink and increasing the land sink. The magenta line and light-pink shaded area show the corresponding mass balance estimates from GCP2016. In each panel the line and equation shown represent the sum of the *x* and *y* variables, and thus the line has a slope of −1, and any deviation perpendicular to the line indicates disagreement on the sum. Here we use the total flux which by mass balance is the whole-atmosphere growth rate (see text), and for panels **(a)** and **(d)**, the total flux – FF line also equals O + L, while for panels **(b)** and **(c)**, the total flux line equals O + L + FF. Ellipses denote the variability around the model means of 1*σ* (darker gray) and 2*σ* (lighter gray). **(a)** Ocean versus land; **(b)** ocean versus land + FF; **(c)** ocean + land versus FF; **(d)** total flux versus − 1 × FF.

Figure 2c and d show the vertical gradients and fluxes for 2009–2011 winter (JFM) and summer (JAS). The agreement between the models and HIPPO observations is not as strong as for annual means. The vertical gradient in the NET winter is reasonably well reproduced by nine models with differences lower than 0.36 ppm. The ACTM-IEA inversion is an outlier and overestimates by 0.94 ppm the winter season average vertical gradient. For ACTM, the global annual IEA emissions are less than CDIAC (Fig. 4c and d), which results in a weaker northern extratropical sink (Figs. 2s and 3a) that corresponds with a more positive LT–UT northern extratropical gradient (Figs. 2a and S2) and a more positive N–S gradient (Fig. S2), comparing just the two ACTM versions. Differences across inversion systems in Fig. S2 also depend on the transport and inversion scheme and the resulting spatial distribution of sources and sinks.

There are generally larger differences between observed and modeled vertical gradients in Northern Hemisphere summer (JAS), with only two models (ACTM-IEA and CAMS) within observation error bars, but the whole range of values is only 0.75 ppm. In this case a linear relationship (*r*^2^ = 0.4) is found between the modeled vertical gradient and the retrieved T+SET fluxes, but not for the NET flux. There is a significant relationship between HIPPO and the land-only zonal average vertical gradient and both are correlated with the T+SET fluxes (Fig. S7), but with a slope of 2.16 ppm yr PgC^1^ for HIPPO while it is 0.93 ppm yr PgC^1^ over land where the vertical gradients are bigger. This suggests that transport errors may be more critical in the summer season or that other factors compensate to obscure the relationship for these relatively coarse time averages in other seasons and for the annual means. While additional insights into model behavior could be gained from more detailed comparisons to individual models or in more controlled inversion ensembles, the varied nature of these inversion systems makes detailed analyses more challenging and beyond the scope of our current study.

For the annual means and winter there are no statistical relationships between the vertical gradients and the retrieved fluxes. This suggests that Northern Hemisphere vertical mixing errors do not play a major role in biasing the flux estimation across these models. However, the retrieved fluxes can still be biased because of the transport errors.

One potential limitation in our analysis could be the use of similar meteorological fields from the ECMWF base analysis and forecast cycle, which is the case for 5 out of 10 simulations. A careful comparison of model transport suggests that nudging to a particular reanalysis product does not imply identical tracer transport between the models (e.g., Prather et al., 2008; Locatelli et al., 2015; Orbe et al., 2017). The transport errors arise from resolved advection and heavily parameterized transport schemes such as convection and boundary layer mixing (Locatelli et al., 2015; Orbe et al., 2017; Krol et al., 2018). Qualitatively, we cannot distinguish the CO_2_ vertical gradient from models using ERA-Interim winds from the five other models.

### 3.2 The latitudinal distribution of retrieved land fluxes

In this section, we present the retrieved land flux partitioning between the NET and the T+SET, as shown in Fig. 3 and Table 2. Because the total sink is the sum of T+SET and NET, these lines have a slope of −1 and any deviation perpendicular to the lines indicates disagreement on the total land sink from the GCP2016 estimate. As noted in the previous section, inverse modeling results for the HIPPO period (2009–2011) are remarkably close to one another (Fig. 3a). These results converge on a NET land sink value slightly larger than 2 PgC yr^−1^ (−2.24±0.29 PgC yr^−1^) and a T+SET land sink of −0.38±0.31 PgC yr^−1^. In Fig. 3, multi-model means are represented by blue diamonds and associated error bars are estimated by the standard deviation across models. The 2009–2011 period is marked by a large tropical land sink because of the strong La Nina event of 2011 (Bastos et al., 2013; Poulter et al., 2014). For these 3 years, the models clearly indicate a negative flux over the tropics and SET land. There are also increasing lines of evidence that the rate of deforestation and climate stress over tropics have been moderated in recent decades (e.g., 2000s), compared to the 1990s (Kondo et al., 2018), with a reduced change in tropical forest cover because the decrease in the South American deforestation has been compensated for by an increased Southeast Asian deforestation (Hansen et al., 2013).

In order to place these recent flux estimates in the context of previous studies, we show the flux estimates by the new models that also estimate fluxes for the earlier periods; two models have available outputs for the T3L2 period (1992–1996) and four for the RECCAP period (2001–2004), as shown in Fig. 3b and c. For Jena, one inversion (s85_v4.1) starts in 1985 and is constrained by only 23 atmospheric sites while the other (s04_v4.1) starts in 2004 and uses 59 sites. Interestingly, the difference between s85_v4.1 and s04_v4.1 for 2009–2011 is rather small (Fig. 3a), less than 0.15 PgC yr^−1^.

According to GCP2016, the total land sink in 2009–2011 was around twice as large (around 3 PgC yr^−1^) compared to that for 1992–1996 (around 1.7PgC yr^−1^) and 2001–2004 (around 1.3 PgC yr^−1^). This is due to the combined effect of natural interannual variability as well as a long-term trend (Ballantyne et al., 2012). The retrieved total land fluxes for all study periods appear to be close to the corresponding GCP estimates with most models falling within the GCP2016 1*σ* uncertainty range. For the 2001–2004 period, the newer simulations move fluxes parallel to the GCP line in the direction of a weaker tropical source and a weaker NET sink relative to the original RECCAP estimates. For the 1992–1996 period, one of the two newer simulations shifts fluxes in that same direction, but not as far as suggested by Stephens et al. (2007).

However, we have revisited the Stephens et al. (2007) estimates, by considering the intercept of the regression lines with the aircraft observations rather than the mean of the three models nearest the annual mean observations and evaluating the error using the standard error of the linear regressions. The selection of three models by Stephens et al. (2007) was somewhat arbitrary as they did not directly overlap the observations and did not agree as well as other models seasonally. This new approach relying on the correlated signal from all models leads to a NET flux of −1.7±0.59 PgC yr^−1^ and a T+SET flux of 0.15±0.66 PgC yr^−1^, a similar shift in NET fluxes but only two-thirds of the shift in T+SET fluxes using the Stephens et al. (2007) subset of models, as shown in Fig. 3b.

For the RECCAP period, we used their Group 1 simulations (JENA, LSCE, MACC-II, CT2011_oi, CTE2013) identified in Peylin et al. (2013), four of which assimilated the observations at the sample time as opposed to using monthly means and all of which solved for fluxes at the resolution of the transport model or for small ecoregions over land. The T+SET flux estimate averaged over the RECCAP Group 1 models is 0.34±0.27 PgC yr^−1^. This is nearly identical to the average of the new models from this study (0.34±0.27 PgC yr^−1^; using CTE2016-FT, CTE2017-FT, CT2016, CAMS v16r1, and Jena s85_v4.1). Both estimate slightly positive T+SET fluxes that are only half of the RECCAP all-model average (0.93±0.90 PgC yr^−1^). Our NET land sink estimates using newer models are less than the previous estimates in the original T3L2 and RECCAP studies for the 1992–1996 and 2001–2004 periods. Conversely, our new estimates suggest a change in the T+SET flux towards greater uptake and/or less emission for these periods; we found a decrease in the T+SET land flux by 0.71 PgC yr^−1^ from 0.56±0.32 PgC yr^−1^ for the 1994–2004 period compared to −0.15±0.43 PgC yr^−1^ for the 2004–2014 period (Fig. S9). Then, to obtain a flux estimate less sensitive to year-to-year variability we calculate the fluxes for the full 11-year 2004–2014 period (Fig. 3d), for which we have five model estimates. For this longer period, the model spread is largely reduced, in particular for the NET land fluxes, and again we find near-neutral T+SET land fluxes. Taking all four of the estimation periods together (Table 2) all of our central estimates for T+SET are within 0.4 PgC yr^−1^ of zero. The tropical land fluxes are −0.2±0.3 PgC yr^−1^ for 2009–2011 and 0.0±0.12 PgC yr^−1^ for 2004–2014. This implies a consistent uptake of carbon by intact tropical forests over several decades.

### 3.3 Variation in retrieved global carbon budgets

The global carbon budget partitioning for 2009–2011 is shown for our suite of models and for GCP2016 (river adjusted) in Fig. 4 with the model mean and GCP2016 reported in Table. In every panel of Fig. 4, the light-pink error band shows the constraint imposed by fixing the values to those of GCP2016, and the associated equation is shown on the graph. The pink diamond represents the GCP2016 estimate while the cross and the gray shaded area show the model mean and 1 standard deviation in darker and 2 standard deviations in lighter gray. For the models, the total flux is calculated as the subtraction of the ocean and land sink from the FF emissions. Note that by mass conservation the total flux equals the whole-atmosphere growth rate (WAGR), but that WAGR may differ from the MBL atmosphere growth rate (AGR) defined by surface stations, because of sampling biases or interannual variability in tropospheric mixing or stratosphere-troposphere exchange. GCP uses the MBL AGR (Dlugokencky and Tans, 2018) as an estimate of total flux and assigns uncertainty of ±0.19 PgC yr^−1^ (Le Quéré et al., 2016) for recent decades, with speculation that the relative uncertainty should decrease when averaging multiple years. Note that, even though the CAMS results systematically align with the GCP2016 estimates in Fig. 4, the two are independent, except for the FF and for the atmospheric data that serve to estimate the total flux in GCP2016. By mass balance, the total annual flux must equal the total growth rate integrated over the entire atmosphere, and this is what we refer to as the total flux.

The integrated ocean versus land fluxes are presented in Fig. 4a. The equation for the range of ocean and land fluxes that would match FF and the total flux estimates from GCP2016 is also shown in Fig. 4a. The models and GCP2016 agree well on the ocean flux with a mean of −2.04±0.51 PgC yr^−1^ over the 3 years of 2009–2011. The multi-model mean of the land flux is −2.61±0.42 PgC yr^−1^. The GCP2016 land flux is −3.04±0.5 PgC yr^−1^ and thus overestimates the model mean. The cloud of model ocean versus land flux estimates are rather scattered around the model mean with a correlation coefficient of only 0.51.

To better understand the reasons for these discrepancies, and specifically to investigate how much of the land spread in Fig. 4a is a result of differences in fossil fuel priors, we plotted the ocean flux versus the sum of land and FF emissions in Fig. 4b. This figure shows a tight correlation across models for these two parameters (*r*^2^ = 0.93). Given that prior uncertainties specified in the inversions for ocean fluxes are typically smaller than those for land and fossil emissions are fixed, this implies, for a given ocean and FF flux combination, the models are adjusting the land fluxes while matching CO_2_ observations. While combining land and fossil fluxes together reduces the random scatter, it does not reduce the range of the continental fluxes, illustrating the fact that models do not simply compensate for biases in fossil priors with land fluxes, but rather that ocean fluxes are affected too (Saeki and Patra, 2017). Conversely, we plot the sum of ocean and land fluxes against FF emissions in Fig. 4c. This figure shows that the ocean + land total sink is largely controlled by the prescribed FF emissions. In general, the models use smaller fossil fuel sources than reported in GCP2016. Figure 4d compares the opposite of FF emissions versus the total flux, again defined by subtraction of the land and ocean fluxes from FF. The spread in models is not parallel to the line defined by the GCP2016 budget closure. We hypothesize that models that overestimate fossil emissions prioritize matching the spatial distribution of CO_2_ and thus estimate overcompensating sinks. The spatial patterns of the different FF priors must also play a role, as well as the strength of the atmospheric constraint on annual timescales imposed by the inversion systems.

**Figure 5.**
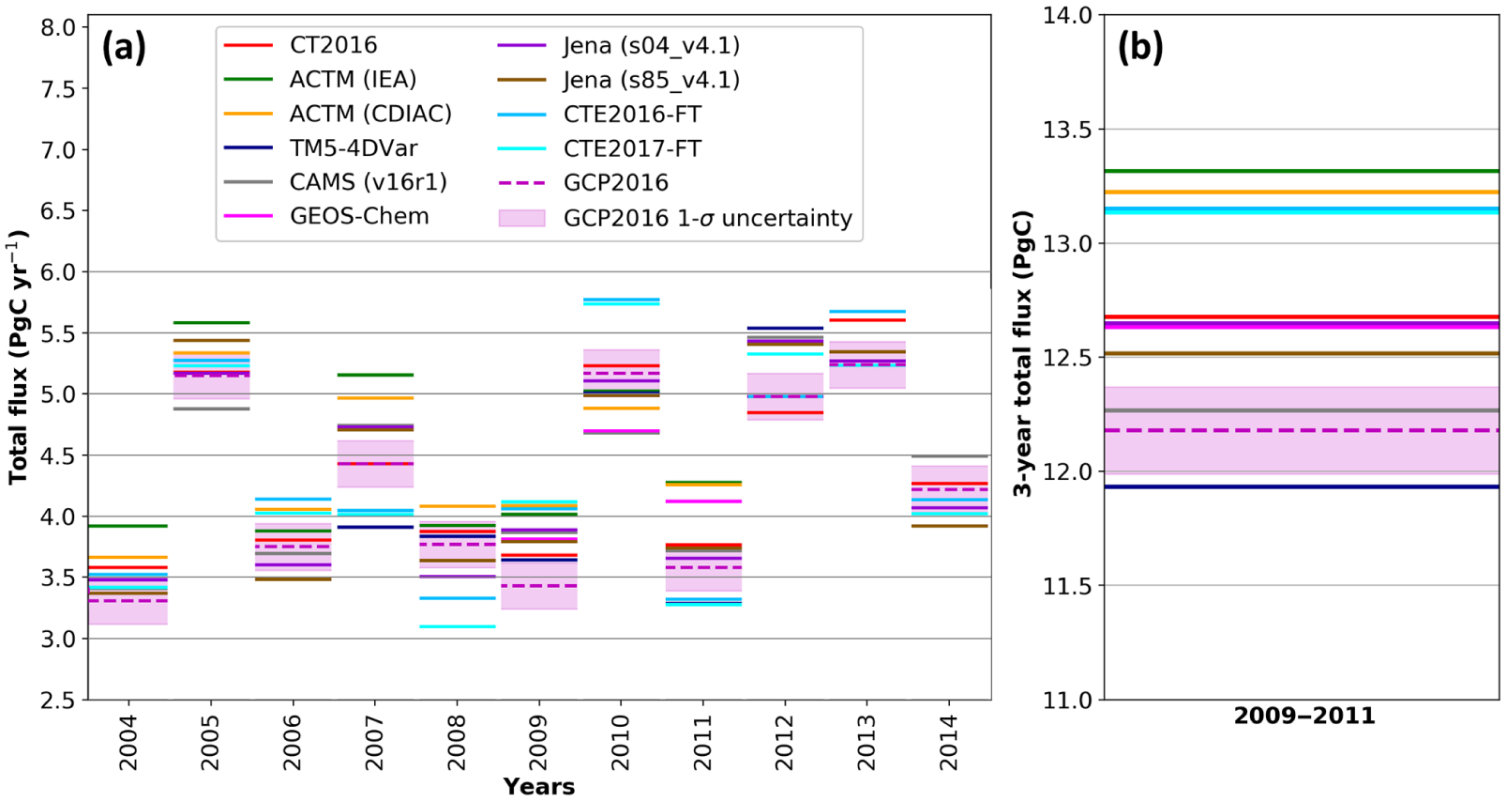
Modeled total flux (lines), equal to whole-atmosphere growth rate, that is the difference between the global FF emissions and the land and ocean fluxes. Atmospheric growth rate from GCP2016, derived from atmospheric CO_2_ measurements made in the marine boundary layer by the NOAA ESRL flask network (Masarie and Tans, 1995; Dlugokencky and Tans, 2018) and GCP2016 assigned uncertainty (pink bands). **(b)** Shows the sum of the total flux for the 3 years (2009 to 2011).

Taking the two extreme models the ACTM-CDIAC and TM5-4DVar estimates provide very different distributions of fluxes. ACTM-CDIAC suggests stronger land sinks, both over the NET and the T+SET regions, and a lower ocean sink while TM5-4DVar suggests the opposite. This leads to a range of around 2 PgC yr^−1^ on the model ocean sink. Because of an intentionally different FF source, but with the same inversion system, the ACTM-CDIAC and ACTM-IEA retrieved land fluxes differ by slightly less than 1 PgC yr^−1^ and ocean fluxes differ by 0.5 PgC yr^−1^. Overall, this analysis suggests that errors in FF priors are larger than the uncertainty prescribed to them or, more specifically, the range of FF estimates used by leading inversions exceeds the uncertainty that GCP2016 places on the CDIAC estimates. This implies that uncertainties in FF emissions do not adequately consider potential regional biases (Peylin et al., 2011; Thompson et al., 2016; Saeki and Patra, 2017). The large spread of model results along the mass balance line in 4C highlights the need (i) to reduce uncertainty in estimates of FF emissions and (ii) to develop modeling systems that relax rigid FF prior constraints and observational systems that can support optimizing FF emission estimates. For the period 1980–2015, the total flux estimates from GCP2016 are estimated by the MBL AGR of Dlugokencky and Tans (2018). Only background sites that are located in the MBL are used in this calculation. Ballantyne et al. (2012) calculated a sampling error of 0.38 PgC yr^−1^ (2*σ*) among the 40 sites and a GCP2017 estimate uncertainty of ±0.19 PgC yr^−1^ (1*σ*) for the period 1980–2015 with respect to the total flux. We show the model-retrieved WAGR (equal to total flux) for each individual year in Fig. 5 along with the GCP2016 estimate and error bars. The total spread in the total flux from the inverse models over the 3 years of 2009–2011 equates to 1.38 PgC as shown in Fig. 5b. This is well outside of the uncertainty range estimated for the extrapolation of MBL measurements, implying several inversions are not rigidly constrained to match observed MBL AGR, even over periods of 3 years. Because CO_2_ is variably mixed in different years and by different models in the troposphere and between the troposphere and the stratosphere, some inconsistency between the MBL-defined AGR and the total flux of CO_2_ in the models might be expected. However, using CT2017 as a test case, the annual difference between the model total surface flux and the observed MBL growth rate over 2000–2016 has a standard deviation of 0.29 PgC yr^−1^ and for 3-year averages within this period a standard deviation of only 0.10 PgC yr^−1^, which is much smaller than the discrepancies shown in Fig. 5. Buchwitz et al. (2018) made a similar AGR comparison using CAMS output of total column and surface data and also found good agreement with differences of only ±0.2 PgC yr^−1^ (1*σ*) on an annual basis. Another potential challenge to inversions having a consistent total flux during this time may be due to large interannual variability in natural fluxes, with rapid changes resulting from different climatic conditions from the moderate El Niño of 2009 to the strong La Niña of 2011 (Bastos et al., 2013; Poulter et al., 2014). This period has also been marked by rapid changes in emissions, related to lower emissions in 2009 during the financial crisis and a rapid increase in 2010 (Peters et al., 2011). However, Fig. 5 does not indicate that the model total flux estimates for the years 2009–2011 are more divergent than other years. Further work investigating these differences is needed but is beyond the scope of this study. In particular, the length of the assimilation window may have an impact. It may also be possible to force the inverse systems to agree, at least within the MBL, with the observationally defined AGR, and this may help to reduce model spread elsewhere.

## 4 Summary and future work

**Figure 6.**
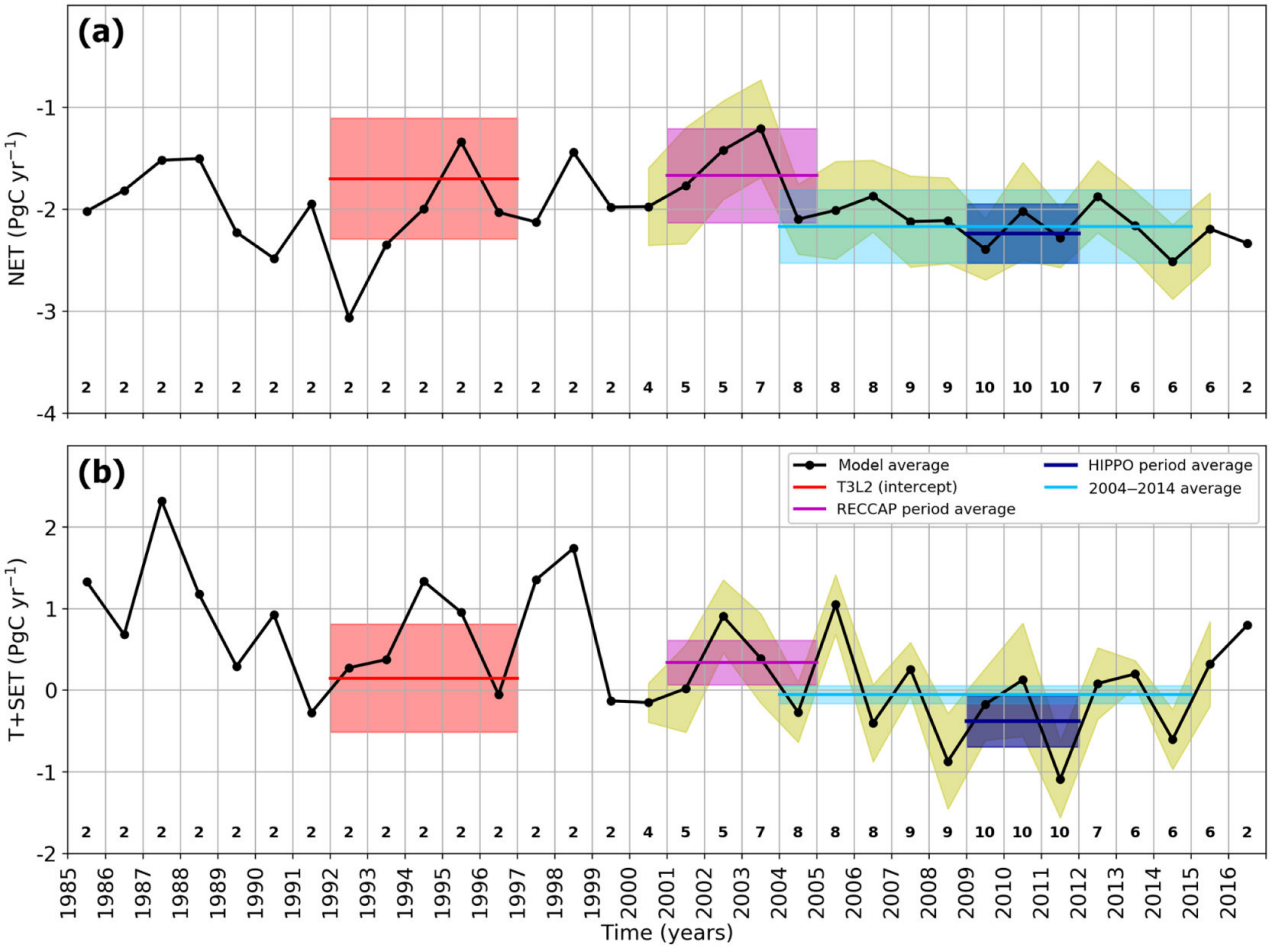
Time series of annual land fluxes for the NET **(a)** and the T+SET **(b)**. The black line represents the model mean and standard deviation derived from available simulations; the number of simulations is shown by the numbers below the curve. The standard deviation is shown only if there are more than two model simulations available. Estimates from the specific period (Table 2) are added as multi-year average and standard deviation (shaded area).

Atmospheric transport has long been a major contributor to top-down atmospheric inverse model flux uncertainty. We applied the technique of Stephens et al. (2007) to a suite of state-of-the-art inversion systems assimilating primarily surface observations to take advantage of the unique HIPPO global airborne dataset for independent validation in assessing fluxes. We also compared the models to each other and to the GCP2016 carbon budget synthesis. The major findings of these comparisons can be summarized as follows:

1. Model estimates of the latitudinal distribution of land fluxes are remarkably consistent across models and this represents a convergence over the past 15 years of inverse model development. The standard deviation across our 10 simulations of the difference between northern extratropical land and tropical land fluxes is 0.4 PgC yr^−1^ for the period 2009–2011 and 0.43 PgC yr^−1^ for the period 2004–2014 across five models. These are considerable reductions from 2.1 PgC yr^−1^ for 12 simulations in T3L2 (differing only in transport modeling) for the period 1992–1996 and 1.28 PgC yr^−1^ for 11 simulations in the RECCAP study for the period 2001–2004.
2. Our suite of 10 inversions gives a NET land uptake of −2.22±0.27 PgC yr^−1^ (1*σ*) and a net T+SET uptake of −0.37±0.31 PgC yr^−1^ for 2009–2011 (−0.2±0.3 PgC yr^−1^ for the tropics only). For 2004–2014, a subset of six models gives NET land uptake of −2.17±0.36 PgC yr^−1^, T+SET uptake of −0.06±0.11 PgC yr^−1^, and T of 0.0±0.12 PgC yr^−1^, thus allowing for deforestation implying a strong uptake in intact tropical forests, in line with forest inventories (Pan et al., 2011).
3. The group of RECCAP models that primarily assimilated discrete rather than monthly mean observations agrees with estimates from our subset of five newer models regarding the lack of strong net emissions from tropical land. This is not too surprising because most of our models, with the exception of LSCEa, are the updated versions of the same models in the RECCAP Group 1 (Peylin et al., 2013). Those five models estimated a net NET land sink of −1.85±0.25 PgC yr^−1^ and our subset of four models covering the RECCAP period estimate of −1.71±0.5 PgC yr^−1^. Regarding T+SET, the newer model estimate is a source of 0.34±0.27 PgC yr^−1^, while it is 0.34±0.27 PgC yr^−1^ in RECCAP’s Group 1.
4. For the 1992–1996 period, we define an update to the Stephens et al. (2007) result, using the intercept of the model output linear regression with the observed annual mean vertical gradient of 0.7 ppm, leading to a NET land uptake of −1.7±0.57 PgC yr^−1^ and a T+SET flux of 0.12±0.62 PgC yr^−1^ for 1992–1996. Our results for the more recent decadal period, the 11 years from 2004 to 2014, indicate a somewhat larger NET sink of 2.21±0.34 PgC yr^−1^ and a neutral tropical land flux of 0.04±0.13 PgC yr^−1^, in line with a trend of a larger land sink (Sarmiento et al., 2010; Keenan et al., 2016) if shared across both latitudinal bands.
5. We present our best estimates of the latitudinal land flux partitioning for the four periods 1992–1996, 2001–2004, 2009–2011, and 2004–2014 in Table 2. We present in Fig. 6 the time series of the NET and T+SET land fluxes from 1979 to 2016, using all simulations available in this study. This figure shows a decrease in the T+SET land flux by 0.71 PgC yr^−1^, from +0.56 to −0.15 PgC yr^−1^ between the decades 1994–2004 and 2004–2014, respectively. The land-use change flux estimated by GCP2017 was nearly identical for these two time periods (+1.31 and + 1.29 PgC yr^−1^, respectively), and assuming these numbers primarily reflect tropical land-use change emissions this implies an increase in the intact tropical forest sink on decadal timescales. Our re-evaluations of the T3L2 and RECCAP study results (Table 2) confirm that the sum of the tropics and southern extratropics have been near neutral for several decades, despite large-scale tropical deforestation, and in accordance with the recent literature on the tropical land carbon budget (Hansen et al., 2013; Keenan et al., 2016;Mitchard, 2018).
6. At the global scale, we find in agreement with earlier studies that our model results are strongly dependent on the prescribed FF emissions. While the total of global land and ocean uptake adjusts to match differences in FF emissions, this compensation is not perfect.
7. Our suite of 10 simulations also retrieve surprisingly different 3-year whole atmospheric growth rates, as defined by the total fluxes. The model range is 1.38 PgC over 3 years, compared to an estimated uncertainty of ±0.10 PgC in CT2017 matching between MBL CO_2_ concentration trends and total flux over 3 years and a 0.2 PgC yr^−1^ uncertainty assigned by GCP2017. The yearly ranges of up to 1 PgC yr^−1^ in the model total flux estimates imply 0.5 ppm disagreements in whole-atmosphere CO_2_ concentrations, and the 1.4 PgC yr^−1^ range for the 3-year period implies disagreements of 0.7 ppm in the whole-atmosphere CO_2_ concentration change over that time period.

Across seven state-of-the-art systems running 10 inversions, there does not appear to be a correlation between posterior NET vertical gradients and the retrieved latitudinal distribution of land fluxes in winter and for the annual mean. This is suggesting that Northern Hemisphere vertical mixing, albeit significant in summer, is not currently the major driver of tropical versus northern extratropical land flux spread. However, transport errors can still contribute significantly to the flux estimates.

Repeating the experiment in T3L2 where transport was the only thing that varied across models would be required to rule out other factors masking a vertical mixing effect, but given the diversity of modern inversion systems this is not practical. Having a common FF prior, eventually also optimized and with known uncertainties, would improve our ability to retrieve the natural fluxes. Other components, such as other features of the transport models, the prior fluxes, or the inversion method also drive the discrepancies in the global atmospheric budget. The ocean, land, and ocean–land partitioning appear to be a function of the FF prior. This also results in large differences in retrieved total flux or WAGR. The increase in the absolute error in fossil fuel emissions and the large sensitivity of the carbon uptake estimates to those errors (Ballantyne et al., 2015) suggest that, despite being thought to be the best-known term in the global carbon budget, systematic errors in fossil fuel emission estimates limit our assessment of the natural fluxes and the ocean–land partitioning from this inversion ensemble.

Our ability to isolate transport effects in this study is limited in comparison to T3L2 in that many other features of the inversion systems also vary; however, this variability allows us to assess the state-of-the-art FF inventories and their importance in the retrieved flux estimates. Gurney et al. (2005), Peylin et al. (2011), and Saeki and Patra (2017) already demonstrated the importance of FF emission uncertainties in inverse modeling studies, suggesting the importance of temporally defined emission inventory. However, Peylin et al. (2011) found that transport errors were still the main source of uncertainty in regional inversions. With the aim of quantifying CO_2_ fluxes at regional scales, it is more than ever necessary to assess systematic errors of inverse modeling results with independent in situ observations. FF emissions could be optimized with the addition of additional species (e.g., Turnbull et al., 2011; Nathan et al., 2018), such as carbon monoxide (Liu et al., 2017; Bowman et al., 2017), although it can be challenging at the most local and urban scales (Ammoura et al., 2016).

There is a significant correlation between NET vertical gradients and the T+SET retrieved fluxes in summer only. This study reaffirms that systematic evaluation of posterior concentrations against independent measurements is essential to assess the biases and accuracy of inverse modeling systems. Future work will naturally involve comparison against CO_2_ observations from the more recent NASA Atmospheric Tomography (ATom) project, which is similar to HIPPO, but is augmented with additional flights over the Atlantic Ocean (see for example Prather et al., 2017) and an extensive atmospheric chemistry payload, and will involve the inclusion of models assimilating satellite total column CO_2_ measurements. It is possible that the larger observation coverage from satellite observations, expanded ^14^CO_2_ measurements, and urban- and power-plant-scale observations will help to narrow down the FF emissions, which in turn will allow us to better evaluate inverse model global and regional land and ocean CO_2_ flux estimates.

## Supplementary Material

Corrigendum

Supplement

## Figures and Tables

**Table 1.** List of the inverse modeling systems used in this study and general characteristics.

Acronym	References	Grid spacing	Fossil fuel priors	Transport model	Number of vertical layers	Meteorological fields	Available period
CAMS (v16r1)	Chevallier et al. (2005, 2010)^a^	3.75° × 1.875°	CDIAC/GCP2016	LMDZ	39	ERA-Interim	1979 to 2016
Jena (s04_v4.1)	Rödenbeck et al. (2003) Rödenbeck (2005)	4° × 5°	CDIAC	TM3	19	NCEP	2004 to 2016
Jena (s85_v4.1)	–	4° × 5°	CDIAC	TM3	19	NCEP	2004 to 2016
CTE2016-FT	van der Laan-Luijkx et al. (2017)	1° × 1°	CDIAC	TM5	25	ERA-Interim	2001 to 2015
CTE2017-FT	–	1° × 1°	CDIAC	TM5	25	ERA-Interim	2000 to 2016
CT2016	Peters et al. (2007)^b^	1° × 1°	ODIAC v2016 and “Miller”	TM5	25	NCEP	2001 to 2015
ACTM-IEA	Saeki and Patra (2017) Patra et al. (2011)	Inversion (2.8° × 2.8°) and forward (1.1° × 1.1°)	IEA	ACTM	32	NCEP2 (for inversion)	2003 to 2011
ACTM-CDIAC	–		CDIAC	ACTM	32	JRA25 for forward	2003 to 2011
TM5-4DVar	Basu et al. (2013)	3° × 2°	EDGAR+CDIAC	TM5	25	ERA-Interim	2007 to 2012
GEOS-Chem	Deng et al. (2014)	4° × 5°	CDIAC, ICOADS and 3-D aviation	GEOS	47	GEOS5	2009 to 2011

a With updates documented at https://atmosphere.copernicus.eu/ (last access: 7 January 2019).

b With updates documented at https://carbontracker.noaa.gov (last access: 7 January 2019).

**Table 2.** Previous and our new best estimates (in bold) of the latitudinal partitioning of land fluxes over four time periods. All values are in PgC yr^−1^. Values are indicated by the model mean±1 standard deviation or 1*σ* error uncertainties. Regarding the T3L2 period (Gurney et al., 2004), our new estimate for the 1992–1996 period comes from the intercept of the fit lines with the observations in Fig. 2b, and the uncertainties on these values come from the standard error on these metrics from the fits. Regarding the RECCAP period (Peylin et al., 2013), our new estimate for the 2001–2004 period is the average of the five new models from this study.

Time period	Source	Number of models	NET land	T+SET land
1992–1996	T3L2	12	−2.42±1.05	0.95±1.17
	Stephens et al. (2007)	3	−1.52±0.53	−0.49±0.25
	T3L2 (intercept)	**12**	−**1.70**±**0.59**	**0.15**±**0.66**
2001–2004				
	RECCAP all models	11	−2.25±0.58	0.93±0.90
	RECCAP Group 1	5	−1.85±0.25	0.34±0.27
	This study	**5**	−**1.67**±**0.46**	**0.34**±**0.27**
2009–2011				
	This study	**10**	−**2.24**±**0.29**	−**0.38**±**0.31**
2004–2014				
	This study	**6**	−**2.17**±**0.36**	−**0.05**±**0.11**

**Table 3.** Global Carbon budget for 2009 to 2011, estimated by the Global Carbon Project 2016 (first row, with river adjustment) and by the suite of models from this study (second row); all values are in PgC yr^−1^. Values are indicated by the model mean±1*σ* error uncertainties, provided by GCP2016 or by the model standard deviation.

	FF	Land	Ocean	Total flux
GCP2016	9.21±0.46	−3.04±0.50	−2.05±0.50	4.06±0.20
Multi-model	8.9±0.29	−2.61±0.42	−2.04±0.51	4.25±0.14
