# Supplementary material for: Global atmospheric CO2 inverse models converging on neutral tropical land exchange, but disagreeing on fossil fuel and atmospheric growth rate
Source: Biogeosciences. Author manuscript; Available in PMC 2020 Jan 16. (PMC6839691; doi:10.5194/bg-16-117-2019)
Supplement: Corrigendum [file NIHMS1029251-supplement-Corrigendum.pdf]

Corrigendum to Biogeosciences, 16, 117–134, 2019
https://doi.org/10.5194/bg-16-117-2019-corrigendum
© Author(s) 2019. This work is distributed under
the Creative Commons Attribution 4.0 License.

Biogeosciences
Open Access

Corrigendum to "Global atmospheric CO_2 inverse models converging on neutral tropical land exchange, but disagreeing on fossil fuel and atmospheric growth rate" published in Biogeosciences, 16, 117–134, 2019

Benjamin Gaubert[1], Britton B. Stephens[2], Sourish Basu[3,4], Frédéric Chevallier[5], Feng Deng[6], Eric A. Kort[7], Prabir K. Patra[8], Wouter Peters[9], Christian Rödenbeck[10], Tazu Saeki[11], David Schimel[12], Ingrid Van der Laan-Luijkx[9], Steven Wofsy[13], and Yi Yin[14]

[1]Atmospheric Chemistry Observations & Modeling Laboratory (ACOM), National Center for Atmospheric Research, Boulder, CO, USA
[2]Earth Observing Laboratory (EOL), National Center for Atmospheric Research, Boulder, CO, USA
[3]Earth System Research Laboratory, National Oceanic and Atmospheric Administration, Boulder, CO, USA
[4]Cooperative Institute for Research in Environmental Sciences, University of Colorado, Boulder, CO, USA
[5]Laboratoire des Sciences du Climat et de l'Environnement, Institut Pierre-Simon Laplace, CEA-CNRS-UVSQ, Gif sur Yvette, 91191 CEDEX, France
[6]Department of Physics, University of Toronto, Toronto, Canada
[7]Climate and Space Sciences and Engineering, University of Michigan, Ann Arbor, MI, USA
[8]RGGC/IACE/ACMPT, Japan Agency for Marine-Earth Science and Technology (JAMSTEC), Yokohama 236 0001, Japan
[9]Meteorology and Air Quality, Wageningen University, Wageningen, the Netherlands
[10]Max Planck Institute for Biogeochemistry, 07745 Jena, Germany
[11]Center for Global Environmental Research, National Institute for Environmental Studies, Tsukuba, Japan
[12]Jet Propulsion Laboratory, California Institute of Technology, Pasadena, CA, USA
[13]School of Engineering and Applied Science and Department of Earth and Planetary Sciences, Harvard University, Cambridge, MA, USA
[14]Division of Geological and Planetary Sciences, California Institute of Technology, Pasadena, CA, USA

Correspondence: Benjamin Gaubert (gaubert@ucar.edu)

Published: 26 March 2019

The authors regret that a typographical error appeared in Table 1 on page 120 of the above-mentioned paper. The CT2016 inverse modeling system uses ERA-Interim as meteorological fields and not NCEP and has a grid spacing of $3° \times 2°$ with a zoom at $1° \times 1°$ over the United States. This information was presented correctly in Sect. S5 of the Supplement. The updated table is shown below.

Published by Copernicus Publications on behalf of the European Geosciences Union.

Table 1. List of the inverse modeling systems used in this study and general characteristics.

Acronym	References	Grid spacing	Fossil fuel priors	Transport model	Number of vertical layers	Meteorological fields	Available period
CAMS (v16r1)	Chevallier et al. (2005, 2010)[a]	3.75° ×1.875°	CDIAC/GCP2016	LMDZ	39	ERA-Interim	1979 to 2016
Jena (s04_v4.1)	Rödenbeck et al. (2003) Rödenbeck (2005)	4° × 5°	CDIAC	TM3	19	NCEP	2004 to 2016
Jena (s85_v4.1)	–	4° × 5°	CDIAC	TM3	19	NCEP	2004 to 2016
CTE2016-FT	van der Laan-Luijkx et al. (2017)	1° × 1°	CDIAC	TM5	25	ERA-Interim	2001 to 2015
CTE2017-FT	–	1° × 1°	CDIAC	TM5	25	ERA-Interim	2000 to 2016
CT2016	Peters et al. (2007)[b]	3° × 2°	ODIAC v2016 and "Miller"	TM5	25	ERA-Interim	2001 to 2015
ACTM-IEA	Saeki and Patra (2017) Patra et al. (2011)	Inversion (2.8° × 2.8°)	IEA	ACTM	32	NCEP2 (for inversion)	2003 to 2011
ACTM-CDIAC	–	and forward (1.1° × 1.1°)	CDIAC	ACTM	32	JRA25 for forward	2003 to 2011
TM5-4DVar	Basu et al. (2013)	3° × 2°	EDGAR +CDIAC	TM5	25	ERA-Interim	2007 to 2012
GEOS-Chem	Deng et al. (2014)	4° × 5°	CDIAC, ICOADS and 3-D aviation	GEOS	47	GEOS5	2009 to 2011

[a] With updates documented at https://atmosphere.copernicus.eu/ (last access: 7 January 2019). [b] With updates documented at http://carbontracker.noaa.gov (last access: 7 January 2019).
