# Supplementary material for: Global atmospheric CO2 inverse models converging on neutral tropical land exchange, but disagreeing on fossil fuel and atmospheric growth rate
Source: Biogeosciences. Author manuscript; Available in PMC 2020 Jan 16. (PMC6839691; doi:10.5194/bg-16-117-2019)
Supplement: Supplement [file NIHMS1029251-supplement-Supplement.pdf]

Supplement of Biogeosciences, 16, 117–134, 2019
https://doi.org/10.5194/bg-16-117-2019-supplement
© Author(s) 2019. This work is distributed under
the Creative Commons Attribution 4.0 License.

Biogeosciences
Open Access

Supplement of

Global atmospheric CO_2 inverse models converging on neutral tropical land exchange, but disagreeing on fossil fuel and atmospheric growth rate

Benjamin Gaubert et al.

Correspondence to: Benjamin Gaubert (gaubert@ucar.edu)

The copyright of individual parts of the supplement might differ from the CC BY 4.0 License.

Supplementary material

1. Calculation of vertical gradients

For all model simulations, we interpolate the models along the HIPPO flight track and mask the output according to the CO2.X availability (2075 out of 28857 10-sec samples missing for our selected flights and regions). Measurements made in the continental boundary layer (Fairbanks missed approach or Anchorage take-off or landing) or in stratospheric air (N_2O < 318 pbb + (year-2009) are excluded. We subtract a deseasonalized smoothed trend component from a seasonal trend decomposition using Loess (STL, Cleveland et al., 1990) fit of the Mauna Loa Observatory in-situ measurement time series to provide a common reference for both observations and models. For the observations and the models, this trend comes from filtering the MLO record with 10-year and 2-year windows and adding these 2 components back together, removing the seasonal and residual terms (see e.g. Stephens et al., 2013).
Then all remaining data are binned in boxes of 5 degrees latitude and 100 hPa altitude. For each box, a 2-harmonic plus annual mean offset fit of the full available time period is performed. An example of this is shown in Fig. S1. For combining individual fit components into larger box means, a weight using the cosine of latitude is applied for each box to more closely match the zonally integrated impact of fluxes. The northern extratropical vertical gradient is calculated as the difference between the average from the 1000 hPa to 800 hPa for the lower troposphere (LT), and the average from 800 hPa to 400 hPa for the upper troposphere (UT), spanning the latitude from 20 N to 90 N. The statistics of the fits for all boxes and for all models and observations are presented in Fig. S2 and S3. Fig. S2 shows the annual mean and Fig. S3 shows the seasonal amplitude. These Figures show that the latitudinal distribution is reasonably well represented in the models while more differences are seen in the vertical.

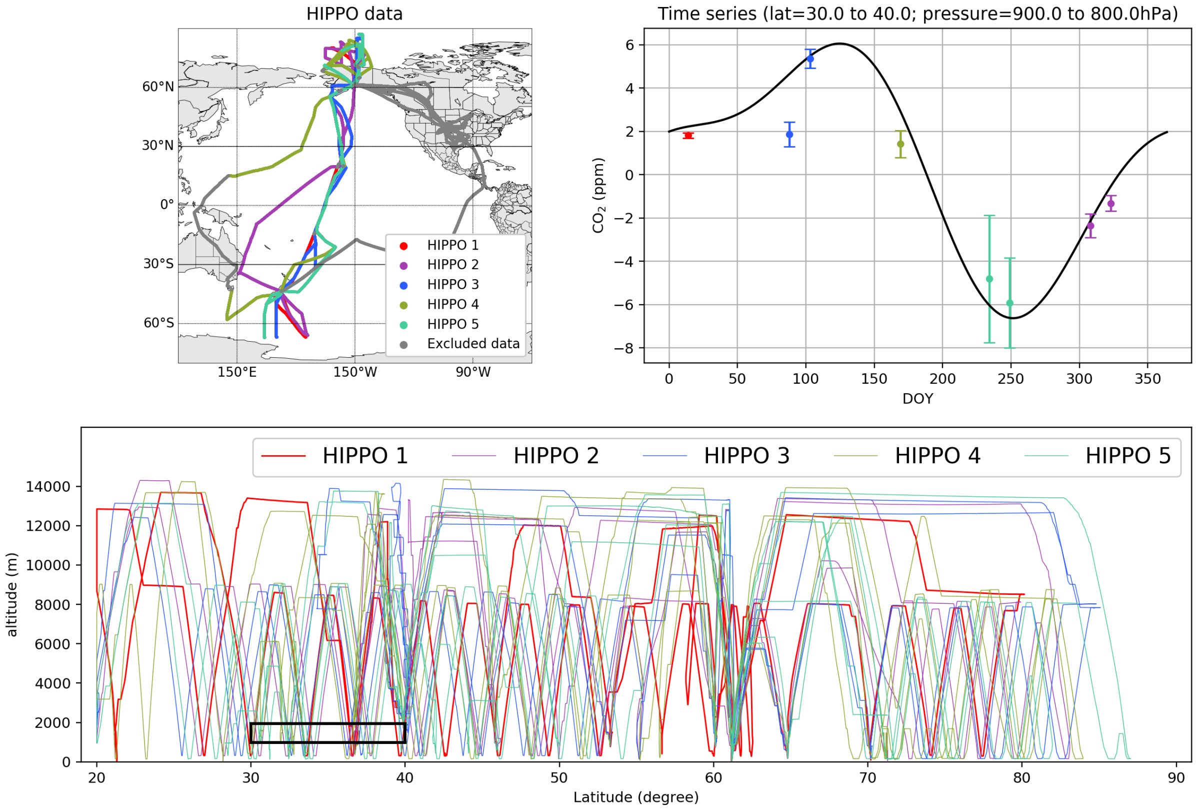

Figure S1: Top left: Map of the data used, coloured by HIPPO campaign number, with grey showing the flight legs excluded. Top right: example of a single fit, with points showing means for each single flight in that specific box (latitude: 30N to 40N, altitude 900 to 800hPa), and standard deviation inside the box represented by error bars. Bottom: altitude versus latitude plot of the CO2.X HIPPO measurements from 20°N to 87°N (note that we only used the > 400 hPa for the analysis).

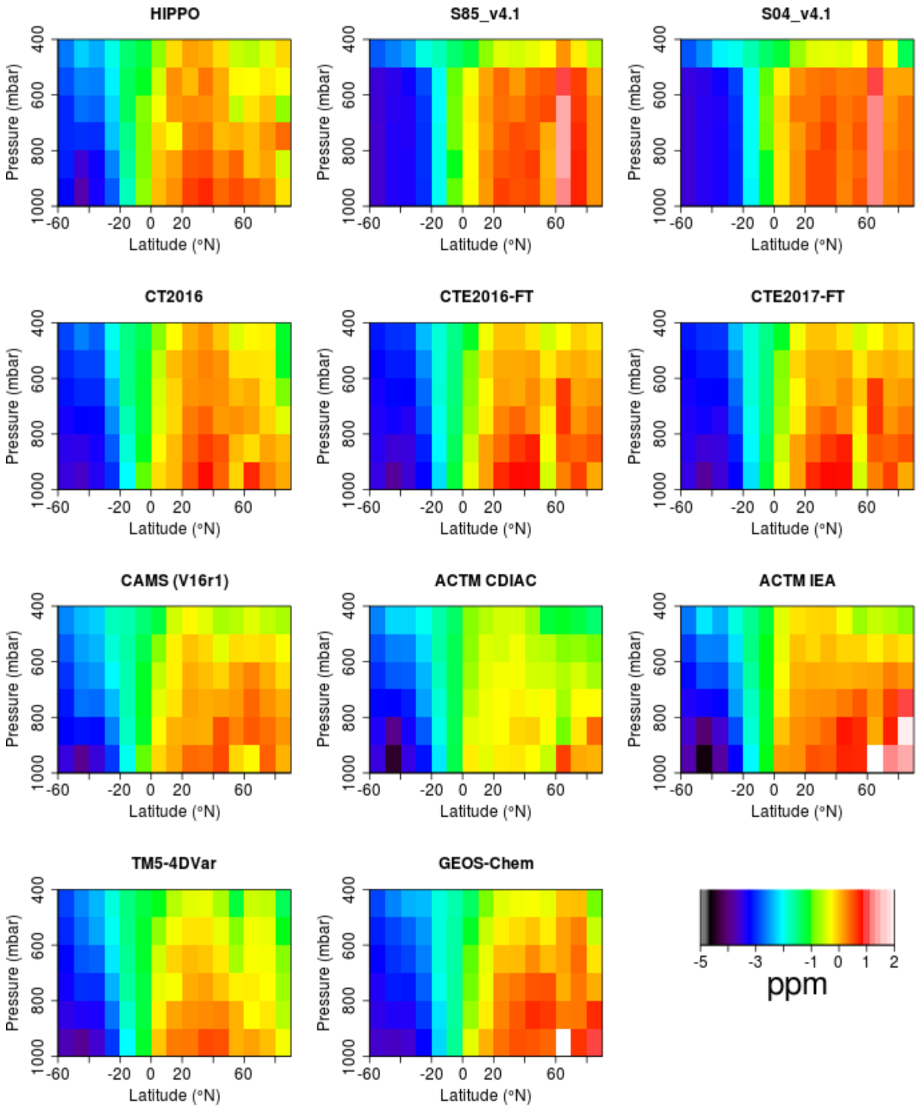

Figure S2: Annual mean CO_2 offsets resulting from the 2-harmonic plus offset fits of the HIPPO data and correspondingly sampled model outputs for every box of 5 degrees latitude and 100 mbar altitude. Note all the models and observations had a deseasonalized smoothed trend component from an STL fit of the Mauna Loa Observatory time series subtracted to provide a common reference for both observations and models.

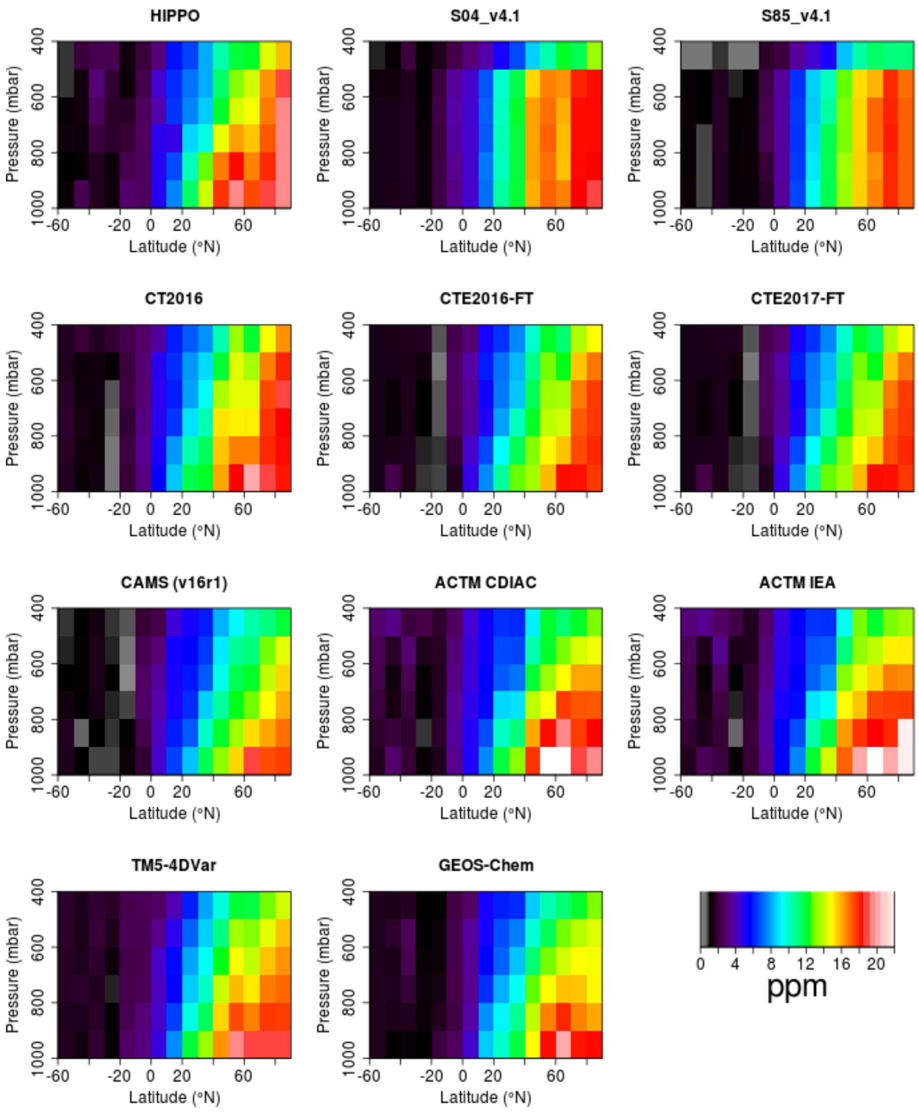

Figure S3: Seasonal amplitude resulting from the 2-harmonic plus offset fits of the HIPPO data and correspondingly sampled model outputs for every box of 5 degrees latitude and 100 hPa altitude.

2. Estimation of measurement uncertainty in vertical gradient

The short-term (10 sec) precision for the QCLS and OMS sensors is on the order of 0.1 ppm and on the order of 0.5 ppm for AO2, while the precision of the flask measurements is also on the order of 0.1 ppm. Thus, the statistical imprecision on vertical gradients defined by many hours of measurements or hundreds of flasks is miniscule. However, airborne measurements may experience altitude-dependent biases for several reasons. Inlet humidity and pressure dependent surface effects can influence both in situ sensors and whole air samplers, cabin pressure changes can affect in situ sensors, and with inadequate drying altitude dependent humidity changes can influence flask measurements.

We assess the uncertainty in the vertical gradient defined by the HIPPO flight tracks, and as calculated in this work, by examining vertical gradients in the differences between the multiple CO_2 sensors and samplers. Because of the wide range of experimental choices on inlet design – pumps, dryers, tubing materials, flow rates, and more – this approach provides a reasonable estimate of our uncertainty in using any single sensor, or combination of sensors, such as the CO2.X variable, which is primarily data from CO2_QCLS with calibration periods gap-filled with CO2_OMS. Model-data mismatch uncertainties specific to the sampling times and coverage of HIPPO are addressed in the next sections.

Table S1 shows the vertical gradients, calculated in several different ways and for different time periods, in the differences between co-located measurements by the different systems. With the 3 in situ sensors, we can calculate vertical gradients identically to how we do so for the CO2.X variable by fitting harmonic functions, as described above. The data density for the whole air samplers is not sufficient to calculate gradients from the same harmonic fitting method as the in-situ data. The in-situ comparisons are shown on the left side of Table S1. As expected, we find close agreement between CO2_QCLS and CO2.X (CO2_QCLS – CO2.X = -0.02 ppm for Ann, not shown in table), but disagreements in the same direction of up to -0.15 ppm for CO2.X compared to CO2_AO2 and CO2_OMS. Recognizing that other common sources of systematic bias might still not be included, we use this full range of disagreement as a conservative bidirectional 1 σ uncertainty estimate of $\pm$ 0.15 ppm in the annual and seasonal vertical gradients and use this as the width of the pink bar in the annual plots in Fig. 2 and S4. If we used CO2_OMS instead of CO2.X our best estimate of the annual mean vertical gradient would be shifted 0.15 ppm to the left in Fig. 1A. For the seasonal plots we use the maximum absolute differences of 0.07 ppm for JFM and 0.17 for JAS for the width of the pink bar.

Using both the in-situ sensors and the whole air samplers, we also calculate the differences in the vertical gradients by simple bin averaging of the sensor differences for each of the 9 transects. For this, we filter for tropospheric N_2O and not continental boundary layer, bin average the CO_2 differences by 10 degrees latitude and 100 hPa bins, and then average these bins with cos(latitude) weighting into larger > 20 N and > 800 hPa, and > 20 N between 400 and 800 hPa bins, to be consistent with our other calculations. We show the vertical differences between these 2 bins on the right side of Table S1. The CO2.X variable is defined at 10 sec resolution but not 1 Hz, and because matching to the Medusa flask sampling kernels requires 1 Hz resolution, we show the difference between CO2_MED and CO2_QCLS rather than CO2.X. We also filter for comparisons with > 75 % overlap between QCLS and Medusa in terms of the kernel weighting. Because CO2.X is defined by CO2_QCLS anywhere that CO2_QCLS exists, these differences are all exactly zero and we do not show them in the table. We use the maximum absolute difference for each transect as conservative bidirectional 1 σ uncertainty estimates and use these to define the size of the vertical error bars for each transect in Fig. 1, with the exception of transect #1 (HIPPO 1 southbound) for which the CCG flask sampler had inadequate drying when we have used the maximum of the other 3 comparisons. The average differences across all 9 transects also support the values determined after harmonic fitting.

	From harmonic fits			From direct averages									
	Ann	JFM	JAS	All trans-ects	H1 SB	H3 SB	H3 NB	H4 SB	H4 NB	H5 SB	H5 SB	H2 SB	H2 NB
Average DOY	-	-	-	-	13	87	104	168	189	233	250	307	324
CO2_AO2 - CO2.X	-0.09	-0.07	0.05	-0.07	0.02	-0.05	-0.13	-0.25	0.14	0.06	-0.10	-0.16	-0.13
CO2_OMS - CO2.X	-0.15	-0.01	-0.17	-0.16	-0.01	-0.07	-0.12	-0.24	-0.23	-0.09	-0.23	-0.23	-0.22
CO2_CCG - CO2.X	-	-	-	-0.13	-1.39	0.07	-0.06	-0.10	-0.03	-0.15	-0.02	-0.33	-0.45
CO2_MED - CO2_QCLS	-	-	-	-0.13	0.00	-0.03	-0.17	-0.25	0.01	-0.05	-0.29	-0.44	0.09

Table S1. Vertical gradients in the CO_2 (ppm) differences between independent sensors and samplers. Calculated as described in the text. The independent transects are shown in seasonal order to match Fig. 1. SB = southbound, NB = northbound.

3. Estimation of potential synoptic model bias

Although the models are all driven by reanalyzed meteorological products, we recognize that there are likely still errors in the positioning of synoptic systems when compared to the real world. To assess how much of the model disagreement we see might be a result of synoptic reanalysis error instead of biased vertical mixing, we have evaluated a single model output over a range of 10 days surrounding the actual HIPPO flights. This represents a worst-case scenario as it simulates a model in which the synoptic systems are completely in the wrong place. We interpolated the Jena s04_v4.1 simulation, on the HIPPO flight track locations but using different time lags, from 5 days before the actual flight and to 5 days after with one day increment. Then, we calculated vertical gradients identically to how we do so for all other models, by binning and fitting harmonic functions as described above. The result is shown in Fig. S4. The standard deviation of all simulations is 0.06 ppm for the annual mean, 0.14 for JFM, 0.15 for JAS. We do not include this worst-case potential model error on our figure, but note that for the annual mean gradient it is considerably smaller than the observational uncertainty, and similar in magnitude to the seasonal uncertainties. This small sensitivity to the positioning of synoptic systems is a result of averaging data over a large latitudinal range. Because synoptic systems typically stir latitudinally, for example in springtime moving high CO_2 air to the south while at the same time moving low CO_2 air to the north, their effect appears to largely cancel out when averaging from 20 to 90 N.

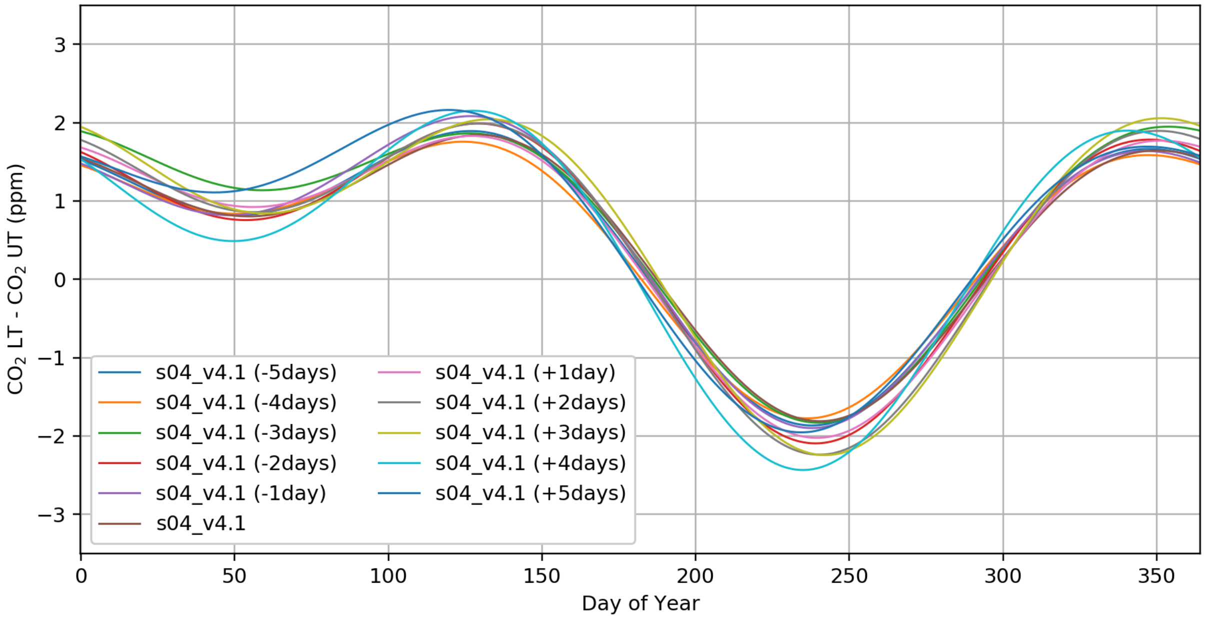

Figure S4: Similar to Fig. 1. Reconstructed annual cycle, obtained from 2-harmonic fits of the Jena s04_v4.1 simulation, NET CO_2 vertical gradients (averaged over 20°N to 90°N and 1000 hPa to 800 hPa minus 800 hPa to 400 hPa). The simulations have been shifted by ± 1 to 5 days around the true time of the observations.

4. Characterization of spatial and temporal representativeness of HIPPO observations

The HIPPO observations provide good vertical coverage but have latitudinal gaps, are mostly over the Pacific Ocean approximately along 150 degrees W, and are for specific months (Fig. S1). This raises the question of the spatial and temporal representativeness of those observations. On Fig. S5, we show the annual mean vertical gradients (2009-2011) for the models at the time and location of the observations on the x axis of panels A and B. From the gridded model outputs, we also calculated the average for the full 150 W transect (Fig. S5A) and for the model zonal mean (Fig. S5B). There is an offset, caused by larger vertical gradients for the zonal mean, which contains more land influence than over 150 W. Overall, despite a larger vertical gradient over land the modelled vertical gradients between the zonal mean, 150 W and sampled along the HIPPO flight track correlate well for the annual means. We show the 150 W vertical gradient on the x axis of panels C and D. The HIPPO vertical gradient is to a certain degree representative of the zonal mean vertical gradients. To evaluate the impact of the time representativeness (Fig. S5D), we look at the zonal mean and 150 W vertical gradients for each individual year (2009, 2010 and 2011). The models show little interannual variability and the relation between zonal means and Pacific data holds.

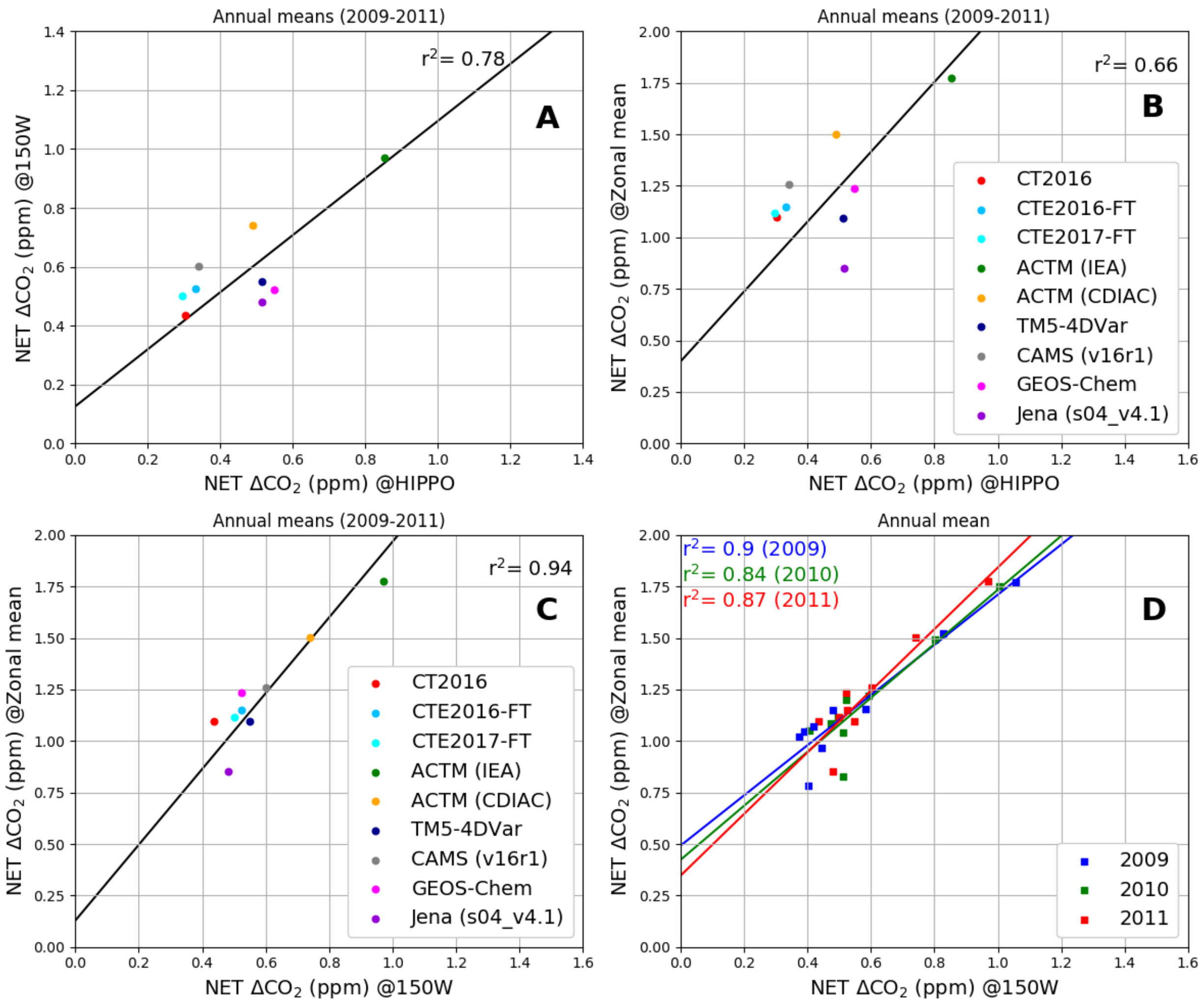

Figure S5: A) Comparison between modelled NET vertical gradient (Δ) at 150 W, versus the reconstructed annual means from the same models sampled coincident with HIPPO observations, during 2009-2011. B) Comparison between zonal mean modelled NET vertical gradient (Δ) versus the reconstructed annual means from the same models sampled coincident with HIPPO observations, during 2009-2011. C) Comparison between zonal mean modelled NET vertical gradient (Δ) and NET vertical gradients at 150 W, during 2009-2011. D) Comparison between zonal mean modelled NET vertical gradient (Δ) and 150 W, separately for each year (2009, 2010 and 2011).

Then, we investigate these spatial relationships, but at monthly and seasonal time scales. Fig. S6A shows all the modelled monthly values over the 3 years between 150 W and the zonal mean. Overall, using all months, the modelled value for the NET 150 W transect correlate with the zonal means. Despite stronger variation than annual means (note the different scales from Fig. S5), the linear relationships in vertical gradients still holds. We also compare the 3-year average retrieved seasonal gradients versus the HIPPO-sampled seasonal gradients. We look at 150 W (Fig. S6B), the zonal means (Fig. S6C) and the zonal means using only land values (Fig. S6D). Significant

relationships exist for JFM, AMJ, and OND for 150 W; for JFM and AMJ for zonal means; and for JAS for zonal means over land.

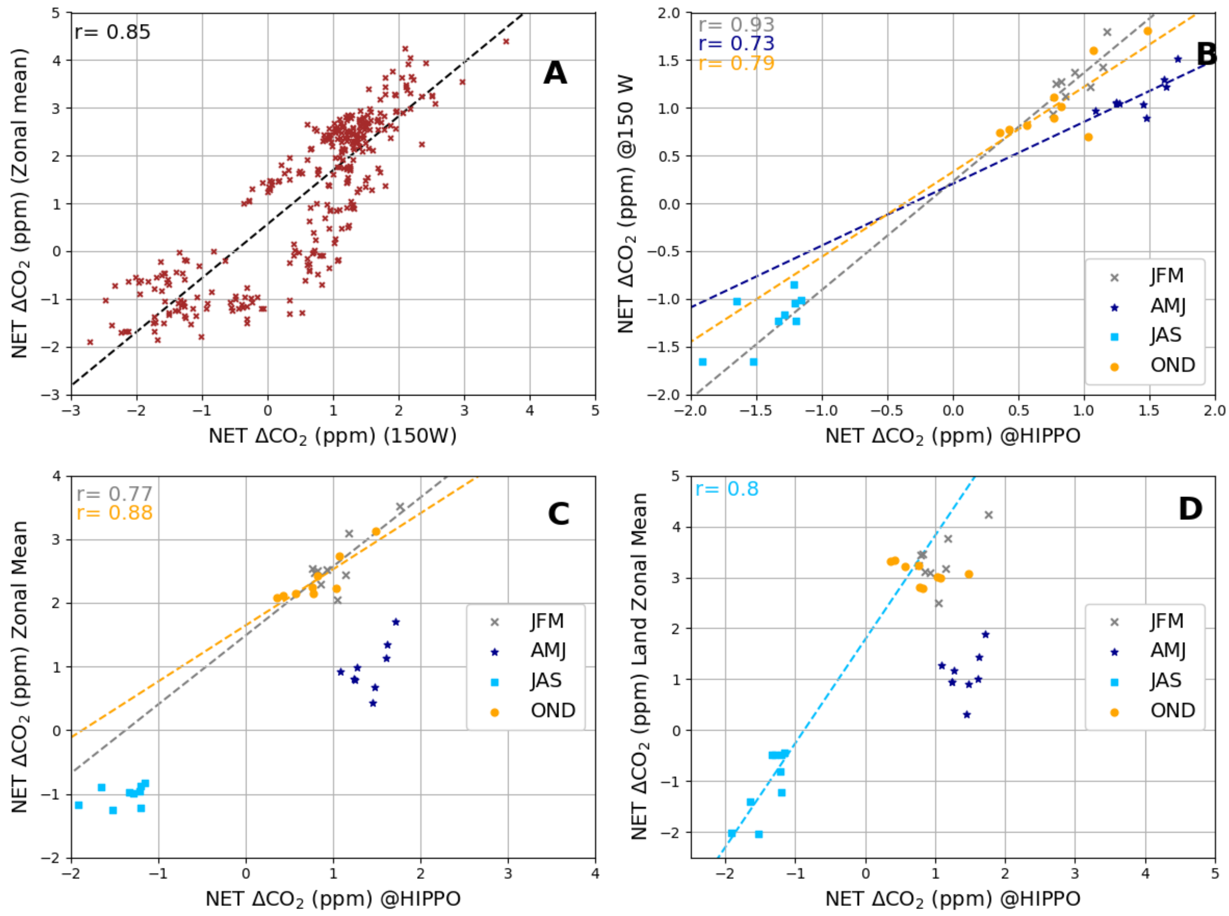

Figure S6: A) Scatter plot of all modeled monthly mean NET vertical gradients for zonal averages versus 150 W . The panels B), C) and D) show seasonal NET vertical gradients modelled over 150W (B), zonal means (C) and zonal mean land only (D) versus the seasonal vertical gradient estimated at the HIPPO flight track locations. When the regression is significant at a 95% level, the line and correlation coefficient are also shown on the graphs.

To assess if we could be missing a flux dependency on transport by only sampling over the ocean, we ask if the vertical gradients were measured over land, would they be correlated with retrieved fluxes? For both seasons and annual means, we compare the NET land and the T+SET fluxes with the vertical gradients, for HIPPO measurements only, over the zonal means, and the zonal means over land only (Fig. S7).

Despite vertical gradients up to 3 times larger, there are still no relationships for all space and time for the JFM season and annual means. However, there is a modest linear correlation between the T+SET land fluxes and the vertical gradients for the HIPPO flight tracks and for the zonal mean averaged over land. This annual and seasonal characteristic is consistent with the findings above, illustrated on the Fig. S5 and S6.

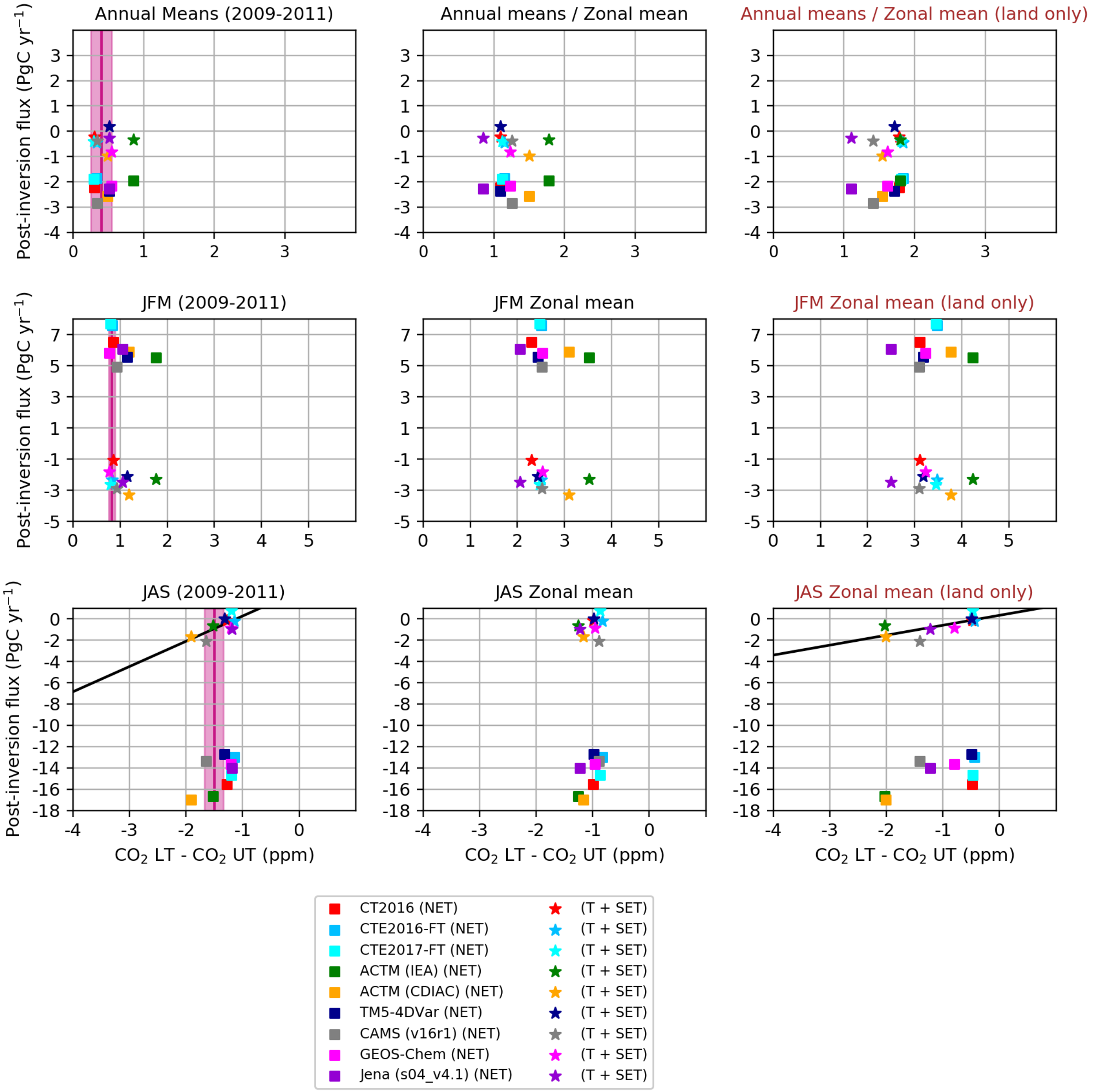

Figure S7: Comparison of the panels (A, C and D) from Fig. 2 (all left panels) with the NET vertical gradients over the zonal mean average (middle column) and the zonal mean over land only (right column). When there is a statistically significant linear relationship, the linear regression line is shown in black.

Finally, we reproduce Fig. 3 from Stephens et al. (2007) substituting T+SET for just T, dividing fluxes at 20 N and 20 S instead of using TransCom regions, and using the 2 newer models that span the 1992-1996 period, CAMS (v16r1) and Jena (S85_v4.1). The CO_2 mole fractions are interpolated at all sites used by Stephens et al. (2007), at 1 km and 4 km at the highest temporal resolution, 3-hourly for CAMS and 6-hourly for Jena. The resulting T+SET fluxes and vertical gradients are presented in Fig. 2 for the annual means and in Fig. S8 for the annual means plus the 2 seasons (JAS and JFM) for 2009-2011. Having only 2 newer models prevents detailed statistical

analysis. It is however worth noting that the JFM vertical gradients are well represented in the newer models and that the annual mean gradient bias appears to arise in summer.

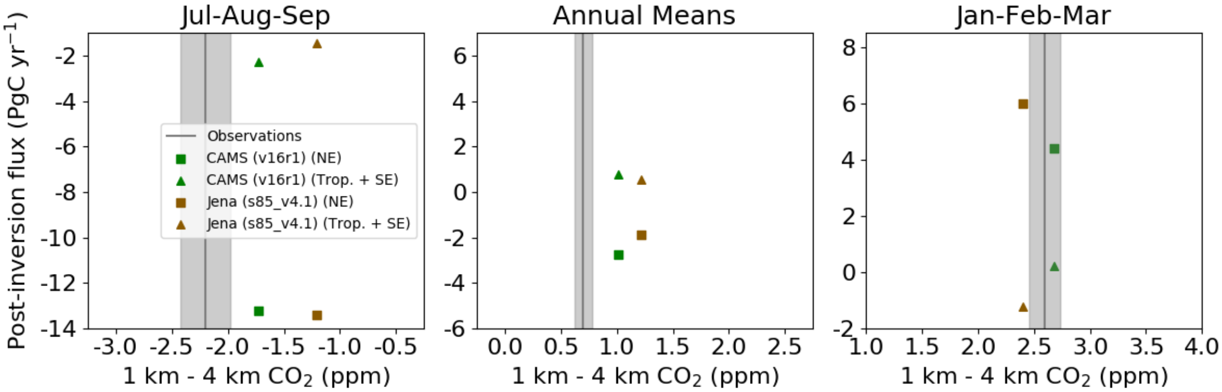

Figure S8: For the period 1992-1996, NET summer (left panel), annual mean (middle panel), and NET winter (right panel) NET (squares) and T+SET (triangles) land carbon fluxes for CAMS v16r1 (green) and Jena s85_v4.1 (brown). The grey line and shaded area represent the observed values (-2.2 ppm for JAS; 0.7 ppm for the Annual Means; 2.6 ppm for JFM) and uncertainty (0.22 ppm for JAS; 0.08 ppm for the Annual Means; 0.14 ppm for JFM) from Stephens et al. (2007).

5. **Description of inversions**

Copernicus Atmosphere Monitoring Service CAMS (V16r1)

Model acronym: CAMS (V16r1)

References: Chevallier et al., 2005; Chevallier et al., 2010

Grid spacing: 3.75° x 1.875°,

Number of vertical levels: 39

Fossil Fuel Priors: EDGAR scaled to CDIAC

Biosphere and Fires Priors: ORCHIDEE (climatology) + GFEDv4

Ocean Priors: Landschüster et al. (2014)

Transport model name: Laboratoire de Météorologie Dynamique with "z" standing for zoom capacity (Hourdin et al., 2006, 2012)

Meteorological fields: European Centre for Medium-Range Weather Forecasts (ECMWF)

Time period (provided): 1979 to 2016

Observations: 119 measurement sites over the globe have been used. Observations were assimilated at their sampled times.

Jena (S04_v4.1 and S85_v4.1)

Model acronym: Jena (S04_v4.1 and S85 v4.1)

References: Rödenbeck et al., 2003; Rödenbeck, 2005; Rödenbeck et al., 2018

Grid spacing: 4° x 5°

Number of vertical levels: 19

Fossil Fuel Priors: EDGAR

Biosphere and Fires Priors: Constant (from LPJ)

Ocean Priors: Mikaloff-Fletcher et al. (2006)

Transport model name: TM3 model (Heimann and Körner, 2003)

Meteorological fields: ERA-Interim (ECMWF, Reanalysis-Interim)

Time period (provided): 2004 to 2016 (S04_v4.1); 1985 to 2016 (s85_v4.1)

Observations: 23 sites (S85v4.1); 59 sites (s04v4.1). Observations were assimilated at their sampled times (flasks) or with hourly averages for continuous measurments, with a correction called "data density weighting" so that the continuous measurements do not dominate the inversion.

Carbon Tracker 2016

Model acronym: CT2016

References: Peters et al., 2007 with updates documented at http://carbontracker.noaa.gov

Grid spacing: 3° x 2° resolution with a zoom at 1° x 1° over the United States.

Number of vertical levels: 25

Fossil Fuel Priors: “Miller" (EDGAR scaled to CDIAC) and "ODIAC"

Biosphere and Fires Priors: Carnegie-Ames Stanford Approach (CASA) biogeochemical model, with GFED 4.1s and GFED_CMS

Ocean Priors: Jacobson et al. (2007) and Takahashi et al. (2009)

Transport model name: TM5 model (Krol et al., 2005)

Meteorological fields: ERA-Interim (ECMWF, Reanalysis-Interim)

Time period (provided): 2004 to 2015

Observations: 66 surface in-situ and a total of 254 number of assimilated observations. hourly average observations are assimilated for continuous measurements, otherwise at their sampled time.

Carbon Tracker Europe 2016 Fast Track and 2017 Fast Track

Model acronym: CTE2016-FT and CTE2017-FT

References: Van der Laan-Luijkx et al., 2017

Grid spacing: 1° x 1°

Number of vertical levels: 25

Fossil Fuel Priors: EDGAR+IER, scaled to CDIAC

Biosphere and Fires Priors: SiBCASA-GFED4

Ocean Priors: Jacobson et al. (2007)

Transport model name: TM5 model (Krol et al., 2005)

Meteorological fields: ERA-Interim (ECMWF, Reanalysis-Interim)

Time period (provided): 2004 to 2015, 2016 for 2017-FT

Observations: 96 sites are assimilated, with hourly averages for continuous measurements.

Transport Model 5 – Four-Dimensional Variational model

Modelling acronym: TM5-4DVar

References: Meirink et al., 2008; Basu et al., 2013

Grid spacing: 6° x 4°

Number of vertical levels: 25

Fossil Fuel Priors: EDGAR+CDIAC

Biosphere and Fires Priors: CASA GFED3

Ocean Priors: Jacobson et al. (2007)

Transport model name: TM5 model (Krol et al., 2005)

Meteorological fields: ERA-Interim (ECMWF, Reanalysis-Interim).

Time period (provided): 2007 to 2012

Observations: 156 sites are assimilated. All observations assimilated were between 12 noon and 4 PM local time, except for mountaintop sites, where the observations were between midnight and 4 AM.

JAMSTEC's Atmospheric Chemistry-Transport Model (ACTM)

Model acronym: ACTM-IEA and ACTM-CDIAC

References: Patra et al., (2009, 2011), Saeki and Patra (2017)

Grid spacing: Inversion T42 (~2.8° x 2.8°); forward run for HIPPO at T106 (~1.1° x 1.1°)

Number of vertical levels: 32

Fossil Fuel Priors: CDIAC-based and IEA/Carbonnes

Biosphere and Fires Priors: CASA-3hourly NEE (Randerson et al., 1997)

Ocean Priors: Takahashi et al. (DSR, 2009)

Transport model name: Atmospheric general circulation model (AGCM)-based Chemistry Transport Model (ACTM)

Meteorological fields: National Center for Environmental Prediction (NCEP)/DOE AMIP-II Reanalysis (Kanamitsu et al., 2002)

Time period (provided): 2003 to 2011

Observations: 66 sites are assimilated as monthly means.

GEOS-Chem

Model acronym: GEOS-Chem

References: Deng et al. (2014); Deng et al. (2015)

Grid spacing: 4° x 5°

Number of vertical levels: 47

Fossil Fuel Priors: CDIAC

Biosphere and Fires Priors: Boreal Ecosystem Productivity Simulator (BEPS) (Chen et al., 1999) GFED3

Ocean Priors: Takahashi et al. (2009)

Transport model name: GEOS, Nassar et al. (2011)

Meteorological fields: Goddard Earth Observing System (GEOS-5) of the NASA Global Modeling and Assimilation Office (GMAO).

Time period (provided): 2009-2011

Observations: 78 sites with continuous observations assimilated at a 1-hour time step.

References:

Basu, S., Guerlet, S., Butz, A., Houweling, S., Hasekamp, O., Aben, I., Krummel, P., Steele, P., Langenfelds, R., Torn, M., Biraud, S., Stephens, B., Andrews, A., andWorthy, D.: Global CO2 fluxes estimated from GOSAT retrievals of total column CO2, Atmos. Chem. Phys., 13, 8695–8717, doi:10.5194/acp-13-8695-2013, 2013.

Chevallier, F., M. Fisher, P. Peylin, S. Serrar, P. Bousquet, F.-M. Bréon, A. Chédin, and P. Ciais, 2005: Inferring CO2 sources and sinks from satellite observations: method and application to TOVS data. J. Geophys. Res., 110, D24309, doi:10.1029/2005JD006390.

Chevallier, F., Ciais, P., Conway, T. J., Aalto, T., Anderson, B. E., Bousquet, P., Brunke, E. G., Ciattaglia, L., Esaki, Y., Fröhlich, M., Gomez, A., Gomez-Pelaez, A. J., Haszpra, L., Krummel, P. B., Langenfelds, R. L., Leuenberger, M., Machida, T., Maignan, F., Matsueda, H., Morgui, J. A., Mukai, H., Nakazawa, T., Peylin, P., Ramonet, M., Rivier, L., Sawa, Y., Schmidt, M., Steele, L. P., Vay, S. A., Vermeulen, A. T., Wofsy, S., and Worthy, D.: CO2 surface fluxes at grid point scale estimated from a global 21-year reanalysis of atmospheric measurements, J. Geophys. Res., 115, D21307, doi:10.1029/2010JD013887, 2010.

Cleveland, R. B., Cleveland, W. S., McRae, J.E. and Terpenning I. (1990) STL: A Seasonal-Trend Decomposition Procedure Based on Loess. Journal of Official Statistics, 6, 3–73.

Daube, B. C., K. A. Boering, A. E. Andrews, S. C. Wofsy.: A high-precision fast-response airborne CO2 analyzer for in situ sampling from the surface to the middle stratosphere. J. Atmos. Oceanic Technol. 19, 10, 1532-1543, 2002.

Dee, D. P., Uppala, S. M., Simmons, A. J., Berrisford, P., Poli, P., Kobayashi, S., Andrae, U., Balmaseda, M. A., Balsamo, G., and Bauer, P.: The ERA-Interim reanalysis: configuration and performance of the data assimilation system, Q. J. Roy. Meteor. Soc., 137, 553–597, https://doi.org/10.1002/qj.828, 2011.

Deng, F., Jones, D. B. A., Henze, D. K., Bousserez, N., Bowman, K. W., Fisher, J. B., Nassar, R., O'Dell, C., Wunch, D., Wennberg, P. O., Kort, E. A., Wofsy, S. C., Blumenstock, T., Deutscher, N. M., Griffith, D. W. T., Hase, F., Heikkinen, P., Sherlock, V., Strong, K., Sussmann, R., and Warneke, T.: Inferring regional sources and sinks of atmospheric CO2 from GOSAT XCO2 data, Atmos. Chem. Phys., 14, 3703–3727, doi:10.5194/acp-14-3703-2014, 2014.

Deng, F., Jones, D. B. A., Walker, T. W., Keller, M., Bowman, K. W., Henze, D. K., Nassar, R., Kort, E. A., Wofsy, S. C., Walker, K. A., Bourassa, A. E., and Degenstein, D. A.: Sensitivity analysis of the potential impact of discrepancies in stratosphere–troposphere exchange on inferred sources and sinks of CO2, Atmos. Chem. Phys., 15, 11773-11788, https://doi.org/10.5194/acp-15-11773-2015, 2015.

Heimann, H. and Körner, S.: The global atmospheric tracer model TM3, Technical Report 5, 13 pp., Max-Planck-Institut für Biogeochemie, Jena, 2003.

Hourdin, F., Musat, I., Bony, S., Braconnot, P., Codron, F., Dufresne, J. L., Fairhead, L., Filiberti, M. A., Friedlingstein, P., Grandpeix, J. Y., Krinner, G., Levan, P., Li, Z. X., and Lott, F.: The LMDZ4 general circulation model: climate performance and sensitivity to parametrized physics with emphasis on tropical convection, Clim. Dynam., 27, 787–813, doi:10.1007/s00382-006-0158-0, 2006.

Hourdin, F., Grandpeix, J.-Y., Rio, C., Bony, S., Jam, A., Cheruy, F., Rochetin, N., Fairhead, L., Idelkadi, A., Musat, I., Dufresne, J.-L., Lahellec, A., Lefebvre, M.-P., and Roehrig, R.: LMDZ5B: the atmospheric component of the IPSL climate model with revisited parameterizations for clouds and convection, Climate Dynamics, 40, 2193–2222, 2012.

Kanamitsu, M., Ebisuzaki, W., Woolen, J., Potter, J., and Fiorino, M.: NCEP/DOE AMIP-II Reanalysis (R-2), B. Am. Meteorol. So., 83, 1631–1643, 2002.

Krol, M., Houweling, S., Bregman, B., van den Broek, M., Segers, A., van Velthoven, P., Peters, W., Dentener, F., and Bergamaschi, P.: The two-way nested global chemistry-transport zoom model TM5: algorithm and applications, Atmos. Chem. Phys., 5, 417– 432, doi:10.5194/acp-5-417-2005, 2005.

Meirink, J. F., Bergamaschi, P., and Krol, M. C.: Four-dimensional variational data assimilation for inverse modelling of atmospheric methane emissions: method and comparison with synthesis inversion, Atmos. Chem. Phys., 8, 6341–6353, doi:10.5194/acp-8-6341-2008, 2008.

Nassar, R., Jones, D. B. A., Kulawik, S. S., Worden, J. R., Bowman, K.W., Andres, R. J., Suntharalingam, P., Chen, J. M., Brenninkmeijer, C. A. M., Schuck, T. J., Conway, T. J., and Worthy, D. E.: Inverse modeling of CO2 sources and sinks using satellite observations of CO2 from TES and surface flask measurements, Atmos. Chem. Phys., 11, 6029–6047, doi:10.5194/acp-11-6029-2011, 2011.

Patra, P. K., M. Takigawa, G. S. Dutton, K. Uhse, K. Ishijima, B. R. Lintner, K. Miyazaki and J. W. Elkins, Transport mechanisms for synoptic, seasonal and interannual SF6 variations and "age" of air in troposphere, Atmos. Chem. Phys., 9, 1209-1225, 2009.

Patra, P. K., Y. Niwa, T. J. Schuck, C. A. M. Brenninkmeijer, T. Machida, H. Matsueda, and Y. Sawa, Carbon balance of South Asia constrained by passenger aircraft CO2 measurements, Atmos. Chem. Phys., 11, 4163-4175, 2011.

Peters, W., Miller, J. B., Whitaker, J., Denning, A. S., Hirsch, A., Krol, M. C., Zupanski, D., Bruhwiler, L., and Tans, P. P.: An ensemble data assimilation system to estimate CO2 surface fluxes from atmospheric trace gas observations, J. Geophys. Res., 110, D24304, doi:10.1029/2005JD006157, 2005.

Peters, W., Jacobson, A. R., Sweeney, C., Andrews, A. E., Conway, T. J., Masarie, K., Miller, J. B., Bruhwiler, L., M. P., Pétron, G., Hirsch, A. I., Worthy, D. E. J., van der Werf, G. R., Randerson, J. T., Wennberg, P. O., Krol, M. C., and Tans, P. P.: An atmospheric perspective on North American carbon dioxide exchange: CarbonTracker, P. Natl. Acad. Sci. USA, 104, 18925–18930, doi:10.1073/pnas.0708986104, 2007.

Rödenbeck, C., Houweling, S., Gloor, M., and Heimann, M.: CO2 flux history 1982–2001 inferred from atmospheric data using a global inversion of atmospheric transport, Atmos. Chem. Phys., 3, 1919–1964, doi:10.5194/acp-3-1919-2003, 2003.

Rödenbeck, C.: Estimating CO2 sources and sinks from atmospheric mixing ratio measurements using a global inversion of atmospheric transport, Technical Report 6, Max Planck Institute for Biogeochemistry, Jena, 2005.

Rödenbeck, C., Zaehle, S., Keeling, R., and Heimann, M.: How does the terrestrial carbon exchange respond to inter-annual climatic variations? A quantification based on atmospheric CO_2 data, Biogeosciences, 15, 2481-2498, https://doi.org/10.5194/bg-15-2481-2018, 2018.

Saeki, T. and Patra P. K, 2017 Implications of overestimated anthropogenic CO2 emissions on East Asian and global land CO2 flux inversion Geosci. Lett. 4 9, doi:10.1186/s40562-017-0074-7, 2017.

Stephens, B. B., Gurney, K. R., Tans, P. P., Sweeney, C., Peters,W., Bruhwiler, L., Ciais, P., Ramonet, M., Bousquet, P., Nakazawa, T., Aoki, S., Machida, T., Inoue, G., Vinnichenko, N., Lloyd, J., Jordan, A., Heimann, M., Shibistova, O., Langenfelds, R. L., Steele, L. P., Francey, R. J., and Denning, A. S.: Weak Northern and Strong Tropical Land Carbon Uptake from Vertical Profiles of Atmospheric CO2, Science, 316, 1732–1735, https://doi.org/10.1126/science.1137004, 2007.

Stephens, B. B., Brailsford, G. W., Gomez, A. J., Riedel, K., Mikaloff Fletcher, S. E., Nichol, S., and Manning, M.: Analysis of a 39-year continuous atmospheric CO_2 record from Baring Head, New Zealand, Biogeosciences, 10, 2683-2697, https://doi.org/10.5194/bg-10-2683-2013, 2013.

Takahashi, T., Sutherland, S. C., Wanninkhof, R., Sweeney, C., Feely, R. A., Chipman, D. W., Hales, B., Friederich, G., Chavez, F., Sabine, C., and Watson, A.: Climatological mean and decadal change in surface ocean pCO_2, and net sea–air CO_2 flux over the global oceans, Deep-Sea Res. Pt. II, 56, 554–577, 2009.

van der Laan-Luijkx, I. T., van der Velde, I. R., van der Veen, E., Tsuruta, A., Stanislawska, K., Babenhauserheide, A., Zhang, H. F., Liu, Y., He, W., Chen, H., Masarie, K. A., Krol, M. C., and Peters, W.: The CarbonTracker Data Assimilation Shell (CTDAS) v1.0: implementation and global carbon balance 2001–2015, Geosci. Model Dev., 10, 2785-2800, https://doi.org/10.5194/gmd-10-2785-2017, 2017.
